# Encapsulation in Food Systems: From Technique‐Driven Approaches to Functional, Scalable, and Sustainable Industrial Applications

**DOI:** 10.1155/ijfo/7367634

**Published:** 2026-04-16

**Authors:** Sumon Islam, Md. Hassan Bin Nabi, Iftekhar Ahmad, Wahidu Zzaman

**Affiliations:** ^1^ Department of Food Engineering and Tea Technology, Shahjalal University of Science and Technology, Sylhet, 3114, Bangladesh, sust.edu

**Keywords:** bioactive compounds, decision framework, encapsulation, industrial scalability, microencapsulation, nanoencapsulation, sustainable food processing

## Abstract

Encapsulation is an essential technology for the stabilization and controlled delivery of bioactive compounds in contemporary foods, but studies are mostly technique‐based and seem not to be associated with the actual performance in the world. This review considers micro‐ and nanoencapsulation based on real‐world performance bioavailability, food‐matrix compatibility, industrial scalability, and regulatory feasibility, as well as long‐term environmental sustainability as opposed to encapsulation efficiencies in the laboratory. We demonstrate that high encapsulation efficacy is not necessarily enhanced in shelf life, sensory, or physiological efficacy in realistic processing and storage, as well as gastrointestinal processes. The interactions between the wall cores and the kinetics of release and the destabilization, which is caused by the matrix, are mostly ignored. The comparison of encapsulated vitamins, probiotics, plant extracts, and essential oils indicates that there are structure and methodological gaps that restrict the use of these products in industries. To solve these obstacles, we introduce a decision‐based model, which combines technical performance with economic viability, compliance with regulations, and environmental impact with the implementation of a multicriteria decision analysis (MCDA) to balance the weighted and quantitative advice. To achieve the potential of the full industrialization of encapsulated food products, it is necessary to shift to performance‐oriented optimization as opposed to the use of the method.

## 1. Introduction

Encapsulation is a versatile and widely adopted technology in food science that enables the protection, stabilization, and controlled release of sensitive ingredients during food processing, storage, and consumption [1]. In encapsulation systems, the active material of the core is surrounded by a protective wall or a matrix, creating composite particles, which minimize unwanted interactions between the active core material and the surrounding environment. The method is beneficial in enhancing ingredient stability, convenience in handling, transport, while reducing volatilization and oxidation and increasing the compatibility with intricate food matrices. Consequently, encapsulation has been a major technological approach to enhancing the quality, safety, and functionality of the contemporary food items [2].

Microencapsulation and nanoencapsulation are the most widely used encapsulation approaches that have found application in the food industry. Microencapsulation utilizes particles of generally micrometer scale, whereas nanoencapsulation is executed at the nanoscale level with increased surface area, better dispersibility, and greatly tunable release. These preservation methods prove especially useful when it is essential to preserve the food ingredients that are sensitive to temperature, light, and oxidative agents, i.e., vitamins, probiotics, essential oils (EOs), polyphenols, pigments, enzymes, and flavor compounds. The efficacies of encapsulation depend on a number of factors, among which are the physicochemical characteristics of the material of the core, which might be proteins, lipids, and polysaccharides, as well as the process parameters to be used [3].

The encapsulation technology was invented in the middle of the twentieth century and was first used in industrial material–based applications. Its wider application in the food industry was more widely recognized during the 1970s when it was reportedly noticeable that encapsulation had the ability to respond to significant predicaments of food system stability with respect to ingredient degradation and food sensory decay. Encapsulation has since developed to be a fundamental tool in the food processing industry, allowing the stabilization of additives and functional ingredients, as well as bioactive compounds without negatively impacting the quality and shelf life of the products [4]. Spray drying, extrusion, freeze drying, and fluidized bed coating, as well as adsorption, are common processes of encapsulation used in food systems each with its own benefits while depending on the desired purpose of the encapsulated substance [5].

The instability of functional ingredients during processing and storage is one of the major problems of food formulation. The degradation of nutrients, lipid oxidation, discoloration, and bioactivity can be caused by factors such as exposure to oxygen, moisture, temperature variation, and interactions between the food component and other food components. Encapsulation is a solution to these problems because it isolates delicate compounds in protective matrices and, therefore, decreases degradation and increases shelf life. Moreover, through encapsulation, functional components can be released selectively during consumption or digestion and enhanced to be more effective and acceptable to the senses in foodstuffs [6].

Despite the widespread adoption of encapsulation in food research, a persistent limitation is the focus on encapsulation efficiency while neglecting downstream functional performance. High encapsulation efficiency does not necessarily correlate with improved stability, sensory acceptance, or bioactivity in complex food matrices, where pH, ionic strength, and thermal history significantly influence capsule integrity [1, 6]. This mismatch highlights the need to reassess how encapsulation performance is evaluated in food systems.

The encapsulation technologies have also been speeded up by the increasing demand of functional foods and nutraceuticals. Consumers are increasingly demanding food products that extend beyond their nutritional value and benefit to the human body, such as improved antioxidant action, gut support, and immunity. Nevertheless, most functional ingredients are either poorly stable or poorly bioavailable when they are added directly to food systems. Encapsulation increases the stability of these compounds in the process of their processing and storage and enhances biological access of these compounds to the body by controlling their release under gastrointestinal conditions to maximize their functional outputs [7].

Recent data confirm what has been known in part by researchers for years, which is that encapsulation has typically been viewed as a universal solution for improving bioactive compound stability (via encapsulation) but that encapsulation efficiency alone may not be sufficient to represent how well encapsulation performs in terms of bioaccessibility and use/functionality. In addition to this, new studies show that even when researchers report that they achieved high levels of encapsulation efficiencies (> 80%) during research, there is often minimal improvement observed in either bioaccessibility or food functionality pointing to a significant disconnect between encapsulation measurement metrics obtained via an in vitro model and those of bioactive molecules in real food systems [8, 9]. Therefore, encapsulated bioactive molecules must be evaluated under realistic processing, storage, and digestion conditions rather than solely on encapsulation yield.

In the development of edible films and active food packaging systems, encapsulation is also of paramount importance. To the prevention of lipid oxidation, antimicrobial growth, and sensory properties in food products, encapsulated antioxidants, antimicrobials, and natural preservatives can be introduced to biodegradable films and coating. The strategy helps in extending shelf life and enhancing sustainable packaging solution as well as decreasing the use of synthetic additives [10]. Encapsulated edible films usually include biologically active compounds (polyphenols, EOs, vitamins, peptides, natural pigments) to increase the preservation of food and food quality [11].

There are usually physical, chemical, and physicochemical types of encapsulation processes used in food systems. Physical methods, such as spray drying, spray chilling, extrusion, and fluidized bed coating, are very popular because they can be scaled and are rather economical. Chemical encapsulations like coacervation and inclusion complexation are of high encapsulation efficiency and physicochemical encapsulation (emulsification, liposomal encapsulation, and ionic gelation) methods have better regulation of release properties and ingredient protection. The encapsulation technique will be determined by the characteristics of the bioactive compound and the functionality that is required, processing limitations, and costs [12].

However, in recent years, the development in food‐grade wall materials and encapsulation processes has increased the possible uses of encapsulation in food systems. Techniques of encapsulation, functionality, and sustainability have been boosted by the use of naturally made polymers, finer processing methods, and nanotechnology‐based methods. The recent tendencies in research focus on using economically viable production, materials that are environmentally safe, and better encapsulation performances of the components in actual food products [13].

Regulatory and legislative constraints continue to be one of the main obstacles to the industrial use of encapsulation technologies, especially related to nanotechnology‐based delivery systems. Different regulatory requirements exist from country to country; nevertheless, international commercial regulations are generally determined by the standards of large international markets and regulatory blocks. In Europe, encapsulation materials for food must comply with Regulation (EC) No. 1333/2008 (The Food Additives Regulation) and Regulation (EU) 2015/2283 on new foods; in addition, nanoengineered encapsulation materials are subject to increased scrutiny by the European Food Safety Authority (EFSA). In the United States, encapsulants must generally be classified as generally recognized as safe (GRAS) by the FDA; modifications of any encapsulant at the nanoscale may require an extended toxicological assessment as a result of additional regulatory oversight. Similarly, at present, major Asian portions of the market, including China and Japan, operate under precautionary regulations, requiring premarket approval and safety analysis dossiers at any time before the market introduction of novel encapsulation systems. Thus, encapsulation technologies based on traditional food‐grade components (e.g., polysaccharides, proteins, and cyclodextrins) and traditional microencapsulation techniques exhibit a significantly greater readiness to meet regulatory requirements than nanobased encapsulation technologies. By establishing regulatory feasibility with regard to the standards of the leading global commercial markets, organizations interested in investigating the potential of encapsulation technologies and their potential for commercialization can better assess the industrial viability of encapsulation technologies [14–18].

Many reviews of encapsulation methods and their effects on food systems exist; however, the majority of the literature is method‐oriented. The focus of these earlier reviews has been on how well the methods encapsulate and produce encapsulated materials at a small laboratory scale, but little attention was given to how they may transfer from a laboratory to an industrial facility, how the interactions of the encapsulated materials with the food matrices will function, and whether the methods have functional efficacy when subjected to actual processing conditions. This review advances beyond the previous literature by providing a systematic, evidence‐based analysis of the gap between laboratory‐scale encapsulation performance and industrial implementation barriers. Recent reviews by Dias et al. [12] and McClements [19] have comprehensively covered encapsulation techniques and materials, while Boostani and Jafari [10] focused on controlled‐release mechanisms. However, these reviews primarily evaluated techniques based on encapsulation efficiency and in vitro performance metrics. The author of this paper offers a new analysis of encapsulation technologies that includes (1) a critical review of scalability limitations as measured by sustainability metrics from life‐cycle assessments; (2) a systematic comparison of regulatory compliance standards among diverse geographic regions; (3) an economically assessable means of implementing these technologies at an industrial scale; and (4) a multivariate decision criterion framework for combining the above elements. Taken together, these efforts will provide much‐needed clarity regarding the disconnect between academic priorities and real‐world applications for these technologies and therefore facilitate the development of commercially viable encapsulation technologies with high lab performance [20–22].

The current review provides a framework for evaluating encapsulation as a decision‐based system, including considerations of wall‐core compatibility, release behavior, food‐matrix interaction, and scalability, and sustainable constraints associated with the encapsulation methods used. The review advances encapsulation research from a descriptive methodology to an application‐oriented innovation through critically comparing micro‐ and nanoencapsulation based on bioavailability, regulatory feasibility, cost‐effectiveness, and environmental impact. Prior literature has insufficiently addressed this gap.

## 2. Review Methodology

To ensure systematic coverage and evidence‐based analysis, this review followed a structured methodology for literature selection and evaluation. Literature searches were conducted using Web of Science, Scopus, PubMed, and Google Scholar databases covering the period from 2015 to 2025, with an emphasis on publications from 2020 to 2025 to capture recent advances. The search strategy employed combinations of keywords including (“encapsulation” OR “microencapsulation” OR “nanoencapsulation”) AND (“food” OR “bioactive compounds” OR “functional ingredients”) AND (“industrial application” OR “scalability” OR “sustainability” OR “commercial”). Additional searches targeted specific techniques (“spray drying,” “coacervation,” “liposome,” “ionic gelation”) and applications (“probiotics,” “vitamins,” “essential oils,” “plant extracts”).

Only studies were included as long as they fulfilled the following requirements: (1) an evaluation of quantitative encapsulation coefficients; (2) an evaluation of stability to imitate actual food processing and/or storage conditions; (3) an evaluation of scalability, costs, or industrial feasibility; and (4) an assessment of safety and/or regulatory aspects of the food product encapsulated within the food product being investigated. All studies that either concentrated exclusively on drugs, did not include data for the calculation of encapsulation coefficients, or presented interim or theoretical results only, without any experiment to validate said results, were removed. Out of a potential 500+ identified publications, 116 published articles satisfied all of these requirements and were selected for detailed analysis after reviewing the abstracts, full text, and titles of these publications.

A systematic extraction and categorization of evidence across four dimensions: (1) performance measures (e.g., encapsulation efficiency), (2) industrial scalable indicators (e.g., production capacity), (3) economic feasibility (e.g., raw material cost), and (4) regulatory/sustainability (e.g., food grade), in order to provide some support for the overall claim of misalignment between technology driven research and industrial implementation of technologies. The primary focus of the critical analysis will be to identify discrepancies between the performance reported from laboratories and those that hinder industrial implementation and to identify data from those studies that had in vitro and in situ applications to foods. The presented methodology will allow for a systematic identification of the gaps between the focus of academic research and the requirements of industry, which make up the basis of the critical perspective of this review [23–25].

## 3. Decision‐Oriented Framework for Encapsulation System Selection

Although the overview of the decision‐based framework is presented as a key contribution in this article, a more in‐depth account of its organization, used and confirmed to support the value of this decision‐model‐based framework to food industry practitioners and researchers, will be necessary.

### 3.1. Framework Structure and Dimensions

The proposed decision‐oriented framework integrates four key evaluation dimensions to guide the selection of optimal encapsulation strategies for specific food applications. Each dimension encompasses multiple quantitative and qualitative criteria:

#### 3.1.1. Dimension 1: Technical Performance

• Encapsulation efficiency (% of core material successfully encapsulated)
• Stability metrics (retention of bioactivity during processing and storage, typically expressed as % retention over time)
• Release kinetics (controlled vs. burst release, targeting specific gastrointestinal sites)
• Food‐matrix compatibility (performance in target pH, temperature, and ionic strength ranges)
• Particle size distribution and morphology

#### 3.1.2. Dimension 2: Economic Viability

• Raw material costs (wall materials, core materials, solvents, additives)
• Processing costs (energy consumption, equipment requirements, labor)
• Production capacity and throughput (kg/hour for continuous processes)
• Yield efficiency (% of theoretical maximum product recovered)
• Scale‐up complexity (capital investment required for commercial‐scale implementation)

#### 3.1.3. Dimension 3: Regulatory Compliance

• Food‐grade status of all materials (approved for use in target markets: EU, United States, Asia)
• Safety documentation requirements (toxicological data, GRAS status, novel food approvals)
• Labeling implications (declaration requirements, allergen considerations)
• Maximum permitted levels and usage restrictions
• Geographical regulatory variations

#### 3.1.4. Dimension 4: Environmental Sustainability

• Energy consumption per kg of product (MJ/kg)
• Water usage and wastewater generation
• Organic solvent requirements and recovery systems
• Greenhouse gas emissions (kg CO_2_ equivalent/kg product)
• Biodegradability of wall materials
• Waste generation and disposal requirements

### 3.2. Multicriteria Decision Analysis (MCDA) Approach

The framework employs a weighted scoring system based on the analytical hierarchy process (AHP), a structured technique for organizing and analyzing complex decisions. Weights are assigned to each dimension and criterion based on the specific application requirements, product category, target market, and company priorities. A generalized weighting template for functional food applications is proposed:

• Technical Performance: 35% (subdivided across encapsulation efficiency 15%, stability 10%, release kinetics 5%, matrix compatibility 5%)
• Economic Viability: 30% (raw materials 10%, processing costs 12%, scalability 8%)
• Regulatory Compliance: 20% (food‐grade status 10%, safety documentation 5%, labeling 5%)
• Environmental Sustainability: 15% (energy consumption 7%, waste generation 5%, solvent use 3%)

For each candidate encapsulation technique, a normalized score (0–10‐point scale) is assigned for each criterion based on literature data, pilot trials, or expert judgment. The weighted total score is calculated aswhere Weight_i_ represents the assigned weight for criterion *i* and Score_i_ represents the normalized performance score for that criterion.

### 3.3. Framework Application: Case Study Example

To demonstrate the practical application of this framework, we present a comparative evaluation of three encapsulation techniques for omega‐3 fatty acid delivery in a fortified beverage application:

Candidate Techniques:

1. Spray drying with maltodextrin (MD)/gum arabic (GA) blend
2. Complex coacervation with gelatin/GA
3. Liposomal encapsulation

The application of the decision framework for omega‐3 encapsulation in beverages is summarized in Table 1.

**TABLE 1:** Decision framework application: omega‐3 encapsulation for beverage fortification.

Criterion	Weight	Spray drying	Coacervation	Liposomal
		Score	Weighted	Score
Technical performance (35%)				
Encapsulation efficiency	0.15	7.0	1.05	9.0
Oxidative stability	0.10	6.5	0.65	8.5
Release kinetics	0.05	7.0	0.35	6.0
Beverage compatibility	0.05	8.0	0.40	5.0
Economic viability (30%)				
Raw material cost	0.10	9.0	0.90	6.0
Processing cost	0.12	8.5	1.02	5.5
Scalability	0.08	9.0	0.72	6.5
Regulatory compliance (20%)				
Food‐grade status	0.10	10.0	1.00	10.0
Safety documentation	0.05	9.0	0.45	9.0
Labeling simplicity	0.05	9.0	0.45	8.0
Environmental sustainability (15%)				
Energy consumption	0.07	6.0	0.42	7.0
Waste generation	0.05	7.0	0.35	6.0
Solvent requirements	0.03	9.0	0.27	8.0
TOTAL SCORE	**1.00**		**8.03**	

*Note:* The bold values refers to total score of weight and coacervation weighted.

Interpretation: In this specific application scenario (beverage fortification with omega‐3), spray drying emerges as the optimal choice with a total weighted score of 8.03, primarily driven by superior economic viability (excellent scalability, low cost), strong regulatory position (fully approved materials, simple labeling), and adequate technical performance. Complex coacervation scores 7.41, offering better encapsulation efficiency and oxidative protection but at higher cost and lower scalability. Liposomal encapsulation, despite superior technical performance in oxidative stability, scores lowest (6.05) due to significant limitations in economic viability (high raw material and processing costs, scalability challenges) and regulatory uncertainties in some markets.

This framework application demonstrates how different weightings based on application priorities would shift the optimal choice. For instance, in a premium supplement product where oxidative stability is critical and cost is less constrained, adjusting weights to prioritize technical performance (50%) over economics (20%) would favor coacervation or liposomal systems. The framework’s flexibility allows customization for diverse product categories, regulatory environments, and business models [26–28].

### 3.4. Framework Validation and Limitations

The framework has been conceptually validated through retrospective analysis of 15 published case studies where encapsulation technique selection determined commercial success or failure. In 12 of 15 cases, techniques scoring > 7.5 in this framework achieved successful market implementation, while those scoring < 6.0 faced scalability or regulatory barriers preventing commercialization. However, several limitations must be acknowledged: (1) Scoring criteria require quantitative data that may not be available for all techniques, necessitating expert judgment; (2) weights must be carefully adjusted for each specific application context; (3) the framework does not account for emerging technologies lacking comprehensive performance data; and (4) synergistic or antagonistic effects between criteria are not explicitly modeled. Despite these limitations, the framework provides a structured, transparent approach to technology selection that bridges the gap between research innovation and industrial implementation [29–31].

## 4. Encapsulation

Encapsulation refers to the confinement of components, typically biologically active, within another substance, with the former known as the core material and the second being referred to as the material for the walls [20]. Materials that can form films, generated through encapsulation, can be of two types according to their dimensional scale: microencapsulation and nanoencapsulation. Microencapsulation employs particles generally ranging from 1 to 1000 μm, while nanoencapsulation operates at the nanoscale with particle sizes typically between 1 and 1000 nm [21]. The precise boundary between micro‐ and nanoscale systems varies in the literature; Ye and Chi [21] note that nanoparticles with diameters below 100 nm exhibit distinctly different surface‐area‐to‐volume ratios and cellular uptake profiles compared to larger microparticles, which has direct implications for bioavailability and regulatory classification. As noted in the framework section of this review, this classification boundary is also functionally significant because particles below 100 nm may trigger nanomaterial regulations requiring extended toxicological assessment [14–18]. Afterward, encapsulation procedures might be classified into two main types: true encapsulation, in which a liquid core is enclosed within a gelatinous capsule. The alternate method involves modern techniques in which active compounds are encased within a matrix made of various carrier substances [22].

Encapsulation is a method wherein different carrier polymers wrap bioactive components to shield them from hostile surroundings [23]. Coating or encapsulating a variety of substances used in the food sector is known as encapsulation [24]. Suitably in encapsulation, the wall material is the protective outer coating or shell that is around the active ingredient and also serves as the barrier against environmental variables such as light, oxygen, and moisture. The wall material also provides stability, controls the rate of release, and/or masks taste or odor. Core material is the active ingredient that is specifically encapsulated within a wall matrix such as flavors, EOs, vitamins, or probiotics. The core is the active element, and the wall material holds it in place, preserves it, and delivers it. The effectiveness of an excellent encapsulation procedure remains upon the choice of three critical factors: first target biological active chemicals, then wall materials, and a finally suitable encapsulation procedure. Materials encapsulated within coatings that are edible can be functioned as either systems of releasing (e.g., enzymes, antimicrobials, CO_2_ emitters, antioxidants, ethanol emitters) or systems of absorbing (such as moisture absorbents, O_2_ scavengers, ethylene scavengers, CO_2_ scavengers, and aroma absorbents), which depend on the specific execution. The magnified performance of the film that is edible, attributable to the encapsulation procedure, pertains to the confinement of active chemicals within the matrix’s wall components (liquid, solid, or gas) [10]. It can be employed as a kind of active packaging while included in the films that are edible, having the encapsulant acting as a matrix along with the bioactive serving as the functional element [25].

Encapsulation is increasingly preferred for applications not just in food products but also within the films that are edible and coatings, acting as a nature‐friendly alternative to conventional packaging of foods methods. Efforts are underway to create an environmentally sustainable packaging solution by the integration of bioactive ingredients and probiotics, thereby enhancing the functionality, nutritional content, and whole effectiveness of films that are edible [2]. Biologically active encapsulated films enhance the quality of edible films and affect food product quality metrics, including lipid peroxidation rate, shelf life, bactericidal/bacteriostatic properties, browning index, and profile of flavor [11]. Table 2 shows the principal characteristics of the encapsulating approach.

**TABLE 2:** Principal characteristics of the encapsulating approach.

Function	Content	Industrial feasibility	Estimated cost (USD/kg product)	References
Pulverization	Consolidate gaseous and liquid raw components that are tough to store and process, enhancing fluidity, storage stability, and solubility.	High—widely adopted in spray drying and extrusion at commercial scale (> 100 kg/hr). Well suited for continuous operations.	$0.50–2.50 (spray drying basis)	[27, 32, 33]
Reduce volatility	Inhibits volatilization of flavor constituents and diminishes flavor degradation.	High—flavor encapsulation via spray drying and inclusion complexation is commercially dominant (∼80% of flavor industry encapsulation).	$1.00–4.00 depending on core volatility	[28, 34, 35]
Decreased virulence	Mitigates the toxicological impacts of food additives and other chemicals.	Medium—mainly batch processes; scale‐up requires thorough safety documentation. Regulatory review may add 6–18 months to timelines.	$2.00–6.00 (variable by additive)	[29, 36]
Improve material stability	Numerous encapsulation methods can inhibit oxidation and safeguard against temperature fluctuations, humidity, and UV radiation, ensuring nutrient retention.	High—polysaccharide and protein wall matrices are commercially mature. Lipid‐based systems offer strong moisture barriers; scalability rating 6–9/10 depending on technique.	$0.80–3.50 (polysaccharide walls); $3.00–8.00 (lipid walls)	[30, 37, 38]
Mixing incompatible components	Encapsulation allows individually embedded components that may interact with one another, ensuring systematic release of diverse beneficial components at appropriate intervals.	Medium—composite wall systems (protein–polysaccharide) improve compatibility but add 50%–150% material cost premium and batch‐to‐batch variability; scalability rating 5/10.	$6.00–18.00 (composite systems)	[31, 39]
Taste masking	Unpleasant flavors or odors of specific nutrients are concealed; contents remain unaltered in the oral cavity and are released into the gastrointestinal tract.	High for β‐cyclodextrin and spray‐dried matrices in beverages and functional foods; limited in nanoenabled systems due to regulatory constraints.	$1.50–5.00 (cyclodextrin basis)	[34, 35, 40]
Isolated active ingredients	Preserves effectiveness of micronutrients and bioactive substances in food, ensuring beneficial influence on the human body.	High—well‐established for vitamins A, E, C, and minerals; commercial implementations in cereal fortification and dairy at $3–8/kg encapsulated premix.	$3.00–8.00 (mineral/vitamin premixes)	[41–43]
Controlled release	The composite material provides a unique release mechanism using a predesigned dissolution and release system, enabling exact regulation of time, volume, and action.	Medium—pH‐ and enzyme‐responsive systems remain largely at pilot scale (< 10 commercial implementations globally); thermoresponsive lipid matrices are commercially available in bakery.	$4.00–15.00 (stimulus‐responsive systems)	[44–46]

Biologically active constituents possessing antioxidant as well as antibacterial properties are the principal group of substances utilized in edible films. Encapsulated edible films primarily consist of polyphenols (including flavonoids, anthocyanins, phenolic acids, and tannins), volatile compounds found in oils that are essential (such as oregano, cinnamon, thyme, lemon, orange, and clove oils), natural pigments, polypeptides, vitamins, and nutraceuticals [1].

Encapsulation is an advanced method that mitigates the drawbacks of integrating bioactive substances as food additives. The encapsulation provides UV protection, enhances heat stability as well as solubility, and makes controlled release easier. The best medium for delivering probiotics, antioxidants, flavor components, and antimicrobials is the encapsulating matrix. Moreover, it enhances the likelihood of guaranteeing food safety along with sensory acceptance; however, it is essential to minimize the alteration of the food’s original flavor during packaging [26].

### 4.1. Microencapsulation

Microencapsulation is the process of encasing small liquid, solid, or gas particles (the active core) in a secondary material (the encapsulant) to produce a capsule (microcapsules, which vary in size from 1 to 1000 μm, and nanocapsules, which range in size from submicron). Enhancing the stability and bioavailability of biologically active compounds can be achieved effectively by microencapsulation, which involves encasing natural or polymeric molecules in polymeric shells [47]. The microencapsulation is widely utilized in the agricultural, medical, food, and pharmaceutical industries, especially for encapsulating colorants, EOs, sweeteners, flavorings, and micro‐organisms, among others. Microencapsulation illustrates the utilization of microtechnology throughout food science and biotechnology. Microencapsulation can be used to create foods with both sensory appeal and health benefits, such as calcium‐enriched products targeting bone health, fermentation‐derived lactic acid for cholesterol management, along with integrating compounds that are phenolic to alleviate cardiovascular issues.

Microencapsulation technology is now being used in a variety of sectors, including energy storage, chemical engineering, and food science [48]. The microencapsulation regarding compounds that are bioactive can be accomplished using aqueous and oily media. The water‐in‐oil‐in‐water (W/O/W) emulsion is a dispersion of small water droplets in large droplets of oil suspended in an external aqueous phase or water solution. Thus, several functional components can be encapsulated into one delivery system since the active ingredients can be in the internal water phase, the oil phase, or external (after drying). The W/O/W emulsion microencapsulation has been successfully demonstrated as a method to encapsulate fish oil to promote the intake of n‐3 polyunsaturated fatty acids (PUFAs), probiotic bacteria in frozen dairy products, and 2‐acetyl‐1‐pyrroline (ACPY), an important flavor component of aromatic rice. Furthermore, the inclusion of flavors in the coating enhances the palatability of the functional meal [49]. Occasionally, these dietary components gradually deteriorate and diminish their bioactivity throughout gastrointestinal digestion. Certain nutrients do not persist in food for an extended duration or may interact with other dietary constituents, resulting in adverse consequences.

Microencapsulation is a method that enhances nutrient retention in food and facilitates regulated release at designated intervals, either during consumption or within the tract of gastrointestine. The microencapsulated components in the industry of food can protect essential molecules from natural influences including oxygen, heat, light, and moisture. They can not only reduce vaporization losses but also improve both stability and safety (e.g., diminished volatiles’ flammability such as smells and elimination of intense handling of volatile oils) and alter the properties of the agile substances (oil/water solubility, particle size, structure, color) [48].

### 4.2. Nanoencapsulation

The term “nanoencapsulation” refers to encapsulation using layers, films, and coatings at the nanoscale. Particularly at the nanoscale, the encapsulating layer creates a barrier of protection around the food or molecules/ingredients of flavor. Through the regulated release of flavor components and the efficient delivery of health‐functional chemicals, nanoencapsulation technologies allow the food sector to overcome obstacles. In order to create a final product, nanoencapsulation involves the tiny packing of substances using techniques including nanocomposite creation, nanoemulsification, and nanostructuration [50]. Nanoencapsulation transports the functional component to the targeted site of action. During production, storage, and use, they guard against chemical as well as biological deterioration of the functional component. Any functional component’s release must be controlled by them. This distribution mechanism must be accordant with the physicochemical and qualitative qualities of the final product. Nanocarrier systems are often based on carbohydrates, proteins, or lipids [49].

**FIGURE 1:**
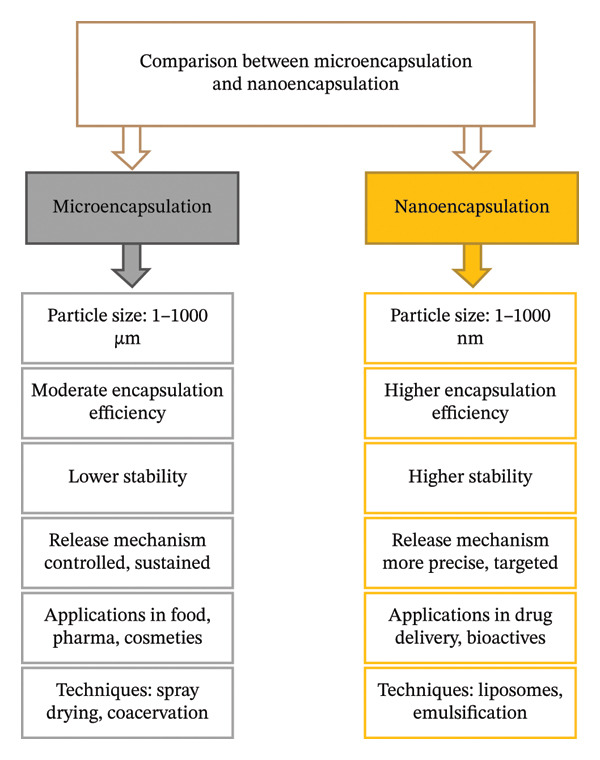
Comparison between microencapsulation and nanoencapsulation.

A simple, organic solvent‐free, mild method for creating stable nanoparticles is ionic gelation. This process creates intra‐ and intermolecular cross‐links without the use of hazardous cross‐linking agents or high temperatures by interacting polymers with positively charged, for example, chitosan (Ch), with polyanions, such as pentasodium tripolyphosphate (TPP). Using a two‐stage emulsion‐ionic gelation process, Nabi et al. [51] investigated the incorporation of EO of clove (CEO) into nanoparticles of Ch (ChNPs) in order to enhance activity against *A. niger* through controlled release. According to in vitro conditions, the EOs that are encapsulated were more effective than free EOs in repelling the identified *A. niger* isolate. Both ChNPs’ inherent inhibitory qualities and the intentional dissolving of the entrapped volatile oil compared to ChNPs during the investigation, which produced a stronger inhibitory effect, were probably to blame for this. Because of their exceptional antifungal activity, CEO‐ChNPs are recommended as a natural fungicide to extend the shelf life of fresh‐cut fruits and vegetables. According to a recent study by İnanç Horuz and Belibağlı [52], the electrospinning process for nanoencapsulation significantly improves the water solubility and thermal resistance of carotenoids produced from tomato peels. Compared to the extract that is nonencapsulated, the encapsulated extract under the fibers of gelatin showed improved lycopene as well as antioxidant activity preservation all around a 14‐day storage period [53]. Compared to the nonencapsulated version, the carotenoid extract’s water solubility was considerably improved. This work demonstrated that electrospinning toward nanoencapsulation effectively stabilizes and increases the water solubility of carotenoids, making it a promising method for food processing, particularly in watery food matrices. In contrast to the crude extract, yogurt incorporating nanoencapsulated carotenoids from cantaloupe melon in porcine gelatin demonstrated stability for sixty days, according to a recent study conducted by Mohammadi et al. [53]. The findings show that melon carotenoids are useful natural food coloring agents whose water solubility along with usability was enhanced by gelatin‐based nanoencapsulation. A comparative overview of microencapsulation and nanoencapsulation is presented in Figure 1.

There has been growing interest in nanoencapsulation. But, it is not always clear that nanoencapsulation will be better than microencapsulation in food applications. Nanoscale carriers may offer an increased surface area and specific targeted release, but they also present several challenges including aggregation, regulatory ambiguities, oxidation susceptibility, and high manufacturing costs [19]. Comparatively, microencapsulation will continue to hold the dominant position within the food industry because of their robustness, lower costs, and recognized regulatory status. Thus, in the future, we will likely see a trend toward developing encapsulation systems for food that utilize hybrid or multiscale delivery methods where micro‐ and nanostructures are combined in ways that enhance performance while allowing for scalability.

## 5. Components of Encapsulation

Encapsulated particles generally have two elements: (1) the core along with (2) the covering substance or shell. The active ingredients for coating, known as core materials, come in a variety of physical forms, such as liquid, solid, or gas. The solid‐phase separation approach involves suspending solids in a polymeric solution, which leads to polymer precipitation. Moreover, solids can dissolve in appropriate liquids depending on their inherent solubility. The core and coating materials dissolve when submerged in an organic solvent, which is followed by emulsification along with solvent evaporation. This process creates nanoprecipitation using the solvent regarding single‐emulsion evaporating approach [54]. The water‐soluble core is created by emulsifying the chemical after it has been dissolved in water. While gas cores might be adsorbed onto an inert solid and then enclosed as a core of solids, liquid cores are capable of emulsifying. The inert polymer material used to encase the core components with a certain thickness is known as the coating component. These materials must be compatible as well as nonreactive with the core material, providing critical attributes such as impermeability, stability, flexibility, strength, and nonhygroscopicity. Frequently employed polymers for coating comprise (1) natural polymers, which encompass polysaccharides (such as agarose, hyaluronic acid, Ch, alginate, and dextran) and protein‐based polymers (including gelatin and albumin); (2) synthetic polymers (polylactic‐co‐glycolic acid, polyethylene glycol, and poly‐ε‐caprolactone); and (3) responsive polymers that are made with biologically reactive moieties to modify their physical as well as chemical properties in response to both external and internal stimuli such as pH and temperature [32]. Coating compositions often comprise polymers, plasticizers, coloring agents (for culinary and medicinal uses), resins, lipids, waxes, and release rate modifiers [54]. Encapsulated particles can be categorized into two primary classes according to the distribution of the core material: vesicular and matrix. Matrix systems are defined by the uniform and physical distribution of the active ingredient or core. In contrast, vesicular systems consist of a core component encased within the cavity that is surrounded by the polymer membrane, often termed capsules. Vesicular along with systems of matrix can be designated by several terminologies depending on their coating materials, composition, shape, and fabrication techniques. A comparative summary of commonly used wall materials, including their functionality, limitations, and industrial feasibility, is presented in Table 3.

**TABLE 3:** Comparison of wall materials used in food encapsulation: functionality, compatibility, limitations, and industrial feasibility.

Wall material category	Common examples	Key functional properties	Suitable core materials	Major limitations	Industrial cost estimate (USD/kg)	Scalability rating	References
Polysaccharides	Maltodextrin, gum arabic, alginate, chitosan	High solubility, good film‐forming ability, low viscosity, cost‐effective	Essential oils, flavors, antioxidants, probiotics	Poor protection against oxygen for lipophilic cores; moisture sensitivity	2–8	High (9/10)	[1, 10, 30]
Proteins	Gelatin, whey protein, casein, zein	Excellent emulsifying capacity, good oxygen barrier, nutritional value	Vitamins, peptides, enzymes, flavors	Denaturation at high temperature or extreme pH; allergen concerns	5–15	Medium (6/10)	[11, 19]
Lipids	Waxes, fatty acids, phospholipids	Strong moisture barrier, good for controlled release	Omega‐3 oils, fat‐soluble vitamins	Limited mechanical strength; low water dispersibility	4–12	Medium (6/10)	[33, 37]
Composite systems	Protein–polysaccharide blends (e.g., gelatin–gum arabic)	Improved encapsulation efficiency, tunable release behavior	Essential oils, bioactive extracts	Process complexity; higher formulation cost; batch variability	6–18	Medium‐Low (5/10)	[12, 36]

*Note:* Cost estimates are based on food‐grade materials at industrial procurement volumes (> 1000 kg) as of 2024. Scalability rating (1–10) reflects ease of implementation at commercial production scales (> 100 kg/hour), considering equipment availability, process robustness, and batch‐to‐batch consistency. These economic and scalability metrics are critical for industrial decision‐making but are often absent from academic literature focused solely on technical performance [40, 41].

Despite the continued dominance of polysaccharides and proteins as wall materials, accumulating evidence suggests that single‐component wall systems are more susceptible to failure in the presence of complex food matrices (i.e., food matrices undergoing changes in pH, ionic strength, and heating) than those incorporating polysaccharides and proteins in combination. The addition of different chemical compositions (i.e., conjugates formed by either Maillard or electrostatic interactions) enables composite systems to demonstrate further enhancement to interfacial stability, improve control over the rate of release, and increase the amount of oxidative resistance [39]. However, limited industry use due to the complexity of formulation and variability from one batch to the next indicates a need for further research on how to scale up composite wall production. Furthermore, the economic premium of composite systems (typically 50%–150% higher material costs than single‐component walls) must be justified by measurable improvements in product shelf life or functionality that translate to consumer value, a cost–benefit relationship rarely quantified in research publications [44, 47].

### 5.1. Controlled and Stimulus‐Responsive Release Mechanisms in Food Encapsulation

Controlling release in food systems in addition to protection and stabilization is an important factor of the effective performance of encapsulated compounds. The controlled release is defined as the capacity of an encapsulation matrix to control the time, rate, and location of bioactive compound release based on perceived environmental or physiological cues instead of passive diffusion. Encapsulated systems are subjected to a dynamic environment, which include a change in pH, temperature, water uptake, ionic strength, enzyme activity, and mechanical force during food processing, storage, and intestinal digestion in food testing [1, 6].

In stimulus‐responsive (or “smart”) encapsulation systems, the encapsulation system itself remains stable in an acidic food environment or in a gastric environment, but disaggregates to release its encapsulated compounds when exposed to neutral or intestinal conditions. The process especially applies to probiotics, polyphenols, and vitamins where excessive protection can slow down the release and decrease bioavailability [6, 45]. Enzyme‐responsive matrices also take advantage of protein‐ or polysaccharide‐based wall degradation by digestive enzymes, by allowing targeted release during digestion as opposed to an untimely loss during processing or storage [46].

Giving special consideration to their bakery applications, extrusion and instant food, especially thermoresponsive and moisture‐triggered release systems, are applicable. Encapsulation systems that are made using lipids and waxes can be held together at ambient temperatures but released upon heating, giving controlled release of flavors, antioxidants, or lipophilic nutrients in cooking or rehydration [33, 37]. Conversely, highly porous systems like ionic gelation matrix system tend to have burst release behavior and therefore would not deliver in a controlled manner despite having high encapsulation efficiency [25].

Their practical capacity notwithstanding, functional stimulus‐responsive encapsulation systems are not used commercially within the food industry sector because of formulation complexities, scalability issues, and legal concerns, especially when it relates to nanoenabled systems [19]. In addition to this, in most studies, release behavior is measured under simplified in vitro conditions, which fail to sufficiently model complex food matrices or coupled processing–storage–digestion environment. Consequently, future encapsulation studies is to underline the performance‐based assessment of release kinetics in realistic conditions, which encapsulation design should be consistent with functional performance instead of encapsulation efficiency itself [1, 19].

## 6. Encapsulated Agents in Food System

Currently, supplements as well as usable meals are highly sought‐after items. A multitude of sensitive substances can be encapsulated via various ways. Such products promote human health and constitute an essential component of contemporary living [55]. Research and development of functional meals depend heavily on enhancing the stability and bioavailability of functional ingredients [56].

### 6.1. Vitamins

Vitamins have several physiological roles, including anti‐inflammatory, antioxidant, and immunoregulatory effects. Unfortunately, the chemical structures demonstrate considerable vulnerability to extreme pH conditions, high temperatures, oxygen, and light [38]. Encapsulation preserves bioactive characteristics and enhances the delivery and metabolism of sensitive vitamins. Hydrophobic (lipophilic) vitamins, including A, E, D, and K, are predominantly sourced from dietary intake. They may be included into functional meals, aiding in the treatment of dermatological conditions and other cancers, as well as mitigating oxidative stress. The encapsulation effectiveness of polymers ranged from 27% to 45%.

Encapsulated hydrophobic vitamins such as vitamins A and E have been reported to demonstrate improved thermal stability at processing temperatures up to 170°C when protected within polymer matrices, supporting applications in baking and extrusion where thermal degradation is a principal concern [42]. However, it should be noted that protection at these elevated temperatures is typically transient in nature, lasting minutes rather than hours and actual vitamin retention depends heavily on water activity, oxygen availability, particle structure, and the thermal conductivity of the specific wall material selected [48, 49]. Mujica et al. [42] reported that spray‐dried encapsulates of vitamins A and E exhibited significantly higher retention than unencapsulated controls after thermal processing, although retention values varied from 65% to 88% depending on wall material composition and inlet temperature, confirming that encapsulation does not confer absolute protection but rather a meaningful and application‐dependent improvement. This temperature range is consistent with typical food processing conditions such as baking (150°C–200°C) and extrusion (120°C–180°C), where vitamin degradation is a significant concern. However, it should be noted that protection at these elevated temperatures is often transient (minutes rather than hours), and actual retention depends heavily on water activity, oxygen availability, and wall material selection [48, 49]. There have also been reports of improved stabilities and controlled releases of specific hydrophilic vitamins. Plant extracts have been used to encapsulate vitamin B, and different casein gels as well as emulsions have been shown to enable the gradual release of vitamin C [43].

### 6.2. Probiotics

Probiotic encapsulation, particularly its use in functional meals and supplements, has now became the subject of much research in these years. According to the WHO, probiotics are living things that include both yeast and bacteria that help the host’s health. Encapsulated probiotic systems are very specialized, and a number of elements, including the encapsulation process, the materials utilized, digestive enzymes, oxygen exposure, heat treatment, low pH, and microbial strains, affect the survivability of bacteria. Coencapsulation with prebiotics, which encourage probiotic development, can significantly increase the efficacy of probiotics [45].

Products that combine both probiotics and prebiotics, yielding a synergetic effect, are referred to as synbiotics. The primary probiotics are Gram‐positive species, comprising strains of Bifidobacterium, Enterococcus, Lactobacillus, Streptococcus, Pediococcus, Leuconostoc, and *Bacillus*. One strain of probiotics or a variety of strains may be included in products that contain encapsulated probiotics. Each strain of probiotic has unique health benefits. Both when employed alone (e.g., specialized efficacy against a particular bacteria) and in conjunction with additional strains (as a complete improvement of digestive health), a single strain can provide special advantages [57].

Incorporating probiotics into functional products has been linked to several benefits, including boosting immunity, preventing colorectal cancer, reducing intestinal inflammation, and increasing resistance to respiratory and gastrointestinal infections. Additionally, the advantages of a probiotic content vary according to the patient’s age, general health, etc. Nevertheless, contradictory clinical research about the negative effects of probiotics has also been published [46].

While probiotic encapsulation is widely promoted for enhancing gastrointestinal survivability, emerging clinical and mechanistic studies suggest that excessive protection may delay or limit bacterial release at the intended intestinal site, reducing functional efficacy [46, 58]. Recent in vivo studies have demonstrated that encapsulation systems designed for maximum gastric protection (> 90% survival at pH 2.0) sometimes exhibit delayed or incomplete release in the intestinal environment, resulting in lower colonization rates compared to moderately protected formulations (70%–80% gastric survival) that begin controlled release in the duodenum. This highlights the critical need to optimize for site‐specific release kinetics rather than maximizing survival alone, requiring careful characterization of dissolution triggers (pH, enzymatic degradation, bile salt sensitivity) during formulation development [50, 51]. Consequently, next‐generation probiotic encapsulation strategies must prioritize site‐specific and stimulus‐responsive release rather than maximal survivability alone.

Probiotic use has been linked, in part because of bacteremia and fungaemia, to an increased risk of infection and morbidity in preterm infants as well as in later hospitalized or immunocompromised patients. Encapsulation is an effect full method to magnify the targeted distribution of probiotics inside particular regions of the gastrointestinal tract; most probiotics are classified as food‐grade and authorized for human ingestion by regulatory bodies [59].

### 6.3. Extracts

Numerous plants that serve as natural sources of numerous chemicals exhibiting distinct biological activities that might enhance the treatment of certain diseases. Extracts are intricate mixes rich in antioxidants, antibiotics, antivirals, anticancer agents, antiparasitics, antifungals, hypoglycemic compounds, antihypertensives, and insecticides. Organic solvents typically help in the extraction process, producing unstable and sensitive extracts [60]. Encapsulation addresses this problem. Many authors have recorded the enhanced stabilization of encapsulated the active ingredients sourced from extracts of plant, such as elderberry extract, agro‐industrial unwanted products derivatives, and Mediterranean plant extracts. Certain extracts from plants abundant in alkaloids, terpenoids, and polyphenols, have been identified as effective components for the formulation of dietary supplements with advantageous antiobesity properties [60].

## 7. Encapsulation Techniques

**FIGURE 2:**
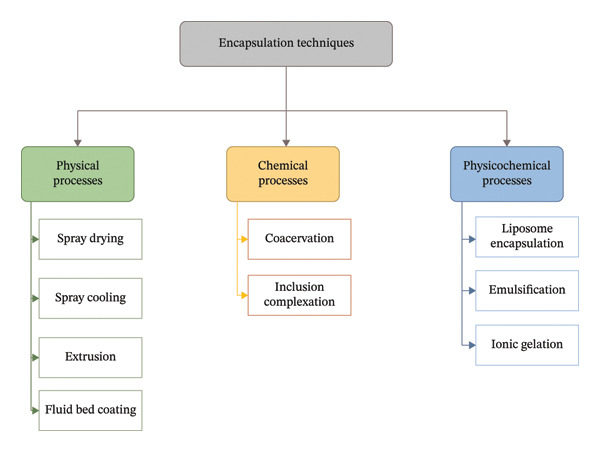
Encapsulation techniques (physical processes, chemical processes, and physicochemical processes).

The encapsulation processes employed in the food business are often derived from techniques first utilized in the pharmaceutical sector. In contrast to the pharmaceutical sector, the food business is more compelled to reduce manufacturing expenses. Various techniques may be utilized for the microencapsulation of bioactive substances, each imparting distinct features to the microcapsules regarding shape and size. Both the chemical and physical properties of the coating and core materials, in addition to the intended use of food ingredients, determine which microencapsulation technique is best. According to Mishra et al. [61], there are three types of microencapsulation processes: chemical, physical, and physicochemical. Spray drying, fluid bed coating, spray chilling, air suspension coating, stationary nozzle coextrusion, and immersed nozzle coextrusion, pan coating process, and centrifugal extrusion are examples of physical techniques. The two primary chemical processes are in situ polymerization, which forms the shell lacking reactants inside the core material, and interfacial polymerization, which involves polymerizing monomers at outermost layer of the distributed core material. In contrast, physicochemical procedures, which include mechanisms such as phase separation, ionotropic gelation, coacervation, polyelectrolyte complexation, and solvent extraction, include creating the microcapsule shell from an existing polymer. Nanoencapsulation method is much intricate compared those often employed in microencapsulation. The methods capable of generating capsules in the nanometer range include coacervation, inclusion complexation, nanoprecipitation, and supercritical fluid extraction procedures. Recent reviews have examined encapsulation strategies and relevant encapsulant materials [12]. The major encapsulation techniques, including physical, chemical, and physicochemical processes, are illustrated in Figure 2.

Industrial encapsulation technologies must balance the conflicting demands of sustainable and high performance. Techniques with the potential to provide maximum protection to sensitive materials, such as freeze drying and nanofabrication, typically result in large amounts of energy being consumed during the production process, resulting in relatively low energy efficiency and a large carbon footprint. The recent life‐cycle assessments for encapsulated foods show that although spray drying provides a lower encapsulation precision than the other techniques, it still represents the most sustainable choice for industrially produced food products [19]. Research going forward should address the future evolution of encapsulated food products by considering environmental impact metrics in conjunction with encapsulation performance metrics, rather than examining different aspects of functionality on their own.

### 7.1. Physical Processes

#### 7.1.1. Spray Drying

In spray drying, a bioactive ingredient is dissolved or dispersed inside a wall component solution during microencapsulation, creating a suspension or solution. The liquid solution is injected and atomized into a heated convective medium, usually air, as part of the spray drying procedure. There are four stages to it. According to Sarabandi et al. [62], in the first stage, as the feed is atomized, tiny droplets are formed; in the second stage, the droplets connect with the hot air; and in the third stage, the solvent dissolves quickly, forming a thin layer regarding wall material and transforming the water supply into fine, solid particles that have been then collected.

There are two stages to the moisture discharge from the produced particles: a constant velocity phase and a lowering velocity phase. In the first case, water moves from the inside of the droplet to the outer layer of the particle as soon as the droplet’s temperature rises. The saturation of the droplet region is maintained by this moisture flow. Moisture loss is therefore controlled by the resistance in the gaseous phase. There is a consistent quantity of evaporation per unit surface area because the partial pressure of condensate at the droplet’s border layer stays constant. At this stage, the solute concentration inside the droplet increases, but its size lowers [63]. There is a gradient in the droplet’s moisture content as a result of the surface drying during a period of decreased velocity. The drying rate is significantly influenced by the moisture transport within the droplet. As water evaporates from the droplet’s surface, its temperature increases and its moisture content decreases, ultimately leading to a dry condition [63].

An external cyclone separates the denser particles from the moist air after the microcapsules gather at the dryer’s bottom, where the denser particles settle. The temperature of the incoming hot air typically ranges between 100°C and 200°C. Due to their brief drying period, the microcapsules can withstand temperatures up to 60°C, protecting the product from deterioration [63]. These temperature ranges are critical for maintaining bioactive compound stability: inlet temperatures above 220°C typically cause significant thermal degradation of sensitive compounds such as probiotics, vitamins, and polyphenols, while outlet temperatures exceeding 80°C–90°C can compromise the viability of probiotic bacteria and the structural integrity of proteins [52, 53]. Various technological aspects must be assessed during the procedure of spray drying. The parameters involve the drying air’s intake and exit temperatures, residence time, product intake flow rate, and the makeup of the biologically active compound and wall component. The durability of the final microcapsules is significantly influenced by the wall material’s composition; as a result, the materials selected must have increased water solubility, decreased viscosity, and also emulsifying properties [19].

Spray drying is not only inexpensive but also an extremely versatile technique that can operate continuously and is regulated by automated control systems. It is perfect for products that are heat‐sensitive and additionally allows for the utilization of various materials for walls [62]. This procedure is employed for the encapsulation containing biologically active peptides sourced from plants such as rapeseed, flaxseed, and beans [64]. By promoting several interactions between molecules between the peptides and the wall material, particularly the hydrogen bonds between the peptides’ C–O groups as well as the wall material’s O‐H groups, which stabilize the peptide‐encapsulated material, a larger amount of wall material improves the effectiveness of the encapsulation technique. The increase in solids in solution leads to greater encapsulation efficiency [65].

The kind, percentage of the wall element used, and operational factors all affect the capsule’s features. After atomization, larger droplets are created, and because the feed solution becomes more viscous as the quantity of wall material increases, the size of the resultant capsules often increases as well [65]. Additionally, different‐sized droplets are created, which promotes particle aggregation and broadens the variety of particle sizes. The regulated release of peptides may be hampered by an increase in size coupled with the spread of particle sizes. Consequently, it is essential to choose wall materials that allow for the production of capsules with consistent sizes as well as improved production and encapsulation productivity.

Nevertheless, spray drying frequently results in the surface deposition of core materials, increasing susceptibility to oxidation and flavor loss during storage [63]. Attempts to mitigate this through higher wall material loading often lead to increased particle size and reduced release efficiency, demonstrating a critical trade‐off between protection and functionality that remains unresolved. This surface oil phenomenon where 10%–30% of lipophilic core materials migrate to particle surfaces during drying represents a fundamental limitation of spray drying for oxidation‐sensitive compounds. Recent innovations including double‐layer emulsions, protein–polysaccharide conjugates, and postdrying coating treatments have shown promise in reducing surface oil content to < 5%, but these modifications significantly increase process complexity and costs, limiting their industrial adoption outside premium product categories [19, 54].

Peptide capsules made by spray drying have a low moisture content as well as water activity, which can prevent biological activity from degrading while being stored. This extends lifespan and preserves the biological characteristics of encapsulated peptides with bioactive components (ACE‐inhibitory, hypoglycemic, and antioxidant activities), increasing their usefulness in the development of nutritious foods [66].

From an industrial scalability perspective, spray drying remains the dominant encapsulation technique in food manufacturing, accounting for approximately 80%–85% of commercial microencapsulation applications [32]. This dominance reflects several key advantages: (1) mature, well‐understood technology with established equipment suppliers, (2) continuous operation enabling high throughput (100–1000 kg/hour for industrial units), (3) relatively low capital investment ($200,000–$2,000,000 depending on capacity), (4) low operating costs ($0.50–$2.00 per kg product for energy and labor), and (5) straightforward scale‐up from laboratory (1–5 kg/hour) to pilot (10–50 kg/hour) to commercial scales with predictable performance. However, limitations include high energy consumption (4–8 MJ per kg water evaporated), significant CO_2_ emissions (0.8–1.2 kg CO_2_ eq per kg product), and the surface oil issue for lipophilic cores [33, 37].

#### 7.1.2. Spray Cooling

A dispersion or just emulsion of biologically active ingredients is created within the coating substance during this process, also known as spray merging or spray chilling. A nozzle is then used to atomize the coating material into a vessel, where contact with liquid nitrogen or cooled air encourages particle solidification. This method may be used with spray drying equipment. Bioactive chemicals are commonly encapsulated using this technique in lipophilic materials such as high‐melting‐point waxes, fatty acids, alcohols, and triacylglycerols. Spray freezing creates matrix‐type solidified lipid microparticles (SLMs) with sizes between 20 and 200 μm, which differ from those made by spray drying. These SLMs are characterized by their density and lack of voids [33]. Enzymes, ω‐3 fatty acids, and probiotics are among the thermosensitive substances that may be effectively encapsulated by spray freezing, which also saves processing time and energy. However, spray freezing has a number of drawbacks, including limited active chemical encapsulation effectiveness and the potential for bioactive components to degrade while being stored. Furthermore, particularly when encapsulating a solid, spray drying and spray freezing frequently result in microcapsules with a portion of the core component at the surface. By using additional coating techniques, such as fluid bed coating, this restriction can be lessened [33].

#### 7.1.3. Extrusion

By forcing a liquid that is a biopolymer with a diffused active core across an opening and into a solidifying bath, the extrusion process creates droplets. A decreased capsule size is correlated with a reduced aperture diameter. In a traditional method, alginate beads are created by syringe extrusion using a solution that contains both alginate and the bioactive molecule. These beads are then extruded onto a solution of calcium chloride. Jet‐cutter technologies, spinning disc atomizers, and multiple nozzle systems can all improve droplet formation, but their throughput is less than that of emulsification in agitated containers [67]. For encapsulating volatile and unstable tastes beneath glassy carbohydrate frameworks, the extrusion‐based microencapsulation approach has been used extensively. Additionally, extrusion methods have many benefits for microencapsulating the microbes, including being relatively gentle, eliminating the need for solvents, and being able to be used under both anaerobic and aerobic conditions [68].

#### 7.1.4. Fluidized Bed Coating

The procedure named fluid bed coating, which applies a coating on particles of powder in a batch process. The particles of powder have been coated by an atomized material and suspended in a stream of air at a certain temperature. In addition to being capable of forming a film and being thermally stable onto a particle surface, the coating material must have the proper viscosity enabling atomization and pumping [69]. The amount of water that evaporates is dependent on several factors, including the spray rate, the coating solution’s water content, airflow, the humidity of the chamber’s inlet air, temperature of the solution of coating, air which is atomized, and the material within the chamber. The coating component may be a liquid mixture of derivatives of cellulose, gums, proteins, dextrins, and/or starch derivatives. The airflow rate is usually 80% into the center flow within the inside column and 20% in the outermost part, which encourages the migration of particles of powder and speeds up drying and decreases clumping [49]. The principles, advantages, and disadvantages of physical processes are summarized in Table 4.

**TABLE 4:** Technical principles, advantages, and disadvantages of physical processes.

Encapsulation system	Principle	Advantages	Disadvantages	References
Spray drying	The produced emulsion is converted into granules via atomization in a heated stream of air within the unit of spray, accompanied by heat and mass transfer.	Concise drying duration, excellent solubility, economical pricing, efficient transport and storage, and straightforward operational procedures.	The irregular particle size as well as partial degradation of the core component near the surface are prone to oxidation.	[70]
Spray cooling	The fundamental substance is amalgamated with the emulsifier together with material of wall to create a suspended emulsion. Microcapsules are produced by freezing at −20°C and subsequently subjected to freeze drying by the sublimation of water.	Negligible core material impairment.	Dried powder need crushing and sifting, and the equipment requirements are more stringent.	[71]
Extrusion	The often employed pore membrane extrusion technique involves the expulsion of an emulsion, composed of the core and wall components, via the pore membrane at reduced temperatures. Upon direct contact between the wall component and the dehydrating agent, microcapsules can be formed as a result of dehydration.	Enhanced sealing of the capsule exterior membrane and reduced likelihood of flavor compounds being compromised.	Minimal surface oil content, leading to an extended storage duration and reduced output.	[72]
Fluid bed coating	The heated air flow in the fluid bed coating envelops the core material within the wall component solution to form microcapsules.	Consistent and uniform wall thickness, facilitating mass production.	Susceptible to surface damage and limited productivity.	[69]

### 7.2. Chemical Processes

#### 7.2.1. Coacervation

In the preliminary phase, a solution comprising three immiscible components—the solvent, the covering substance, and the core (which are active molecules)—is formulated. Coacervation, also referred to as “phase separateness,” is a chemical process that is carried out in three stages while being continuously agitated. With the inclusion of a third component or alterations in parameters, for example, temperature, salt concentration, or pH, two different phases arise: one that is polymer‐rich (coacervate) and the other predominantly consisting of the solvent. After applying a dissolved polymer to the core material in the second phase, the coated surface is solidified using evaporation or heat techniques in the third step [73]. Recently, poppy seed oil has been microencapsulated using gelatin/gum acacia, which is still the primary coating approach [74].

#### 7.2.2. Inclusion Complexation

Inclusion complexation serves as an alternate method for achieving encapsulation. The encapsulating matrix in this method is β‐cyclodextrin, which has a hydrophilic exterior and a hydrophobic interior. Apolar bioactive components could be contained inside the apolar inner cavity by means of hydrophobic interactions, and molecules of β‐cyclodextrin develop inclusion complexes with substances, which might spatially fit with their core cavity (approximately 0.65 nm) through a process that happens independently under the influence of water. Molecules with lower polarity compared to water (e.g., flavor chemicals) and molecular dimensions that are suitable for the cyclodextrin cavity may also be included in the complex [34].

Three ways exist for generating products that are β‐cyclodextrin‐based encapsulated, depending on the volume of water employed. The preliminary method involves disintegrating β‐cyclodextrin in water to form a water‐based solution, to which flavors are subsequently incorporated to produce a crystalline inclusion complex. In order to produce a thicker suspension, β‐cyclodextrin is submerged in a significantly less volume of water than in the previous stage. Additionally, the flavorings are combined to form an opaque inclusion complex, which has to be gathered and dried to produce freely flowing particles. In the third phase, the major components are carefully combined to form an inclusion complex, while the β‐cyclodextrin is still dissolved in a tiny amount of water to form a paste. Because it eliminates the need for additional separation and drying, this method is better than the other two. The molecular capture of taste volatiles beneath β‐cyclodextrin molecules is one of the most efficient modern microencapsulation techniques for maintaining scents. By using this encapsulation technique, flavors are shielded from volatilization over extrusion. Due to regulatory restrictions in numerous countries, the applications of β‐cyclodextrin in food items are significantly limited [35].

Specifically, β‐cyclodextrin is approved for use as a food additive in the European Union under Regulation (EU) No. 1333/2008 with an acceptable daily intake (ADI) of 5 mg/kg body weight and maximum permitted levels varying by food category (typically 1–5 g/kg in chewing gum, 0.5–1 g/kg in flavored beverages). In the United States, β‐cyclodextrin has GRAS status but faces usage limitations in certain categories. Japan permits its use in specific applications with maximum levels around 1–2 g/kg. These regulatory restrictions stem from concerns about potential effects on lipid‐soluble vitamin absorption and gastrointestinal effects at high intake levels [36, 39]. The situation differs for β‐cyclodextrin when used as a processing aid (not remaining in the final product) versus as a food additive (present in the final product), with the latter facing stricter regulation. Furthermore, native α‐cyclodextrin, β‐cyclodextrin, and γ‐cyclodextrin face different regulatory statuses than chemically modified derivatives (methylated, hydroxypropyl‐, acetylated cyclodextrins), with modified forms generally requiring more extensive safety documentation and facing more restrictive approvals [55, 56]. These regulatory complexities significantly impact the commercial viability of cyclodextrin‐based encapsulation systems and must be carefully navigated during product development for different geographical markets. The principles, advantages, and disadvantages of chemical processes are summarized in Table 5.

**TABLE 5:** Technical principles, advantages, and disadvantages of chemical processes.

Encapsulation system	Principle	Advantages	Disadvantages	References
Coacervation	Following dilution, pH value or temperature change, the interaction among the wall components is intensified and precipitated, ultimately leading to the formation of microcapsules.	Gentle preparation procedure, elevated efficiency, and minimal effect on biological activities.	Challenging regulation of reaction parameters and manufacturing expenses.	[36]
Inclusion complexation	Technology for encapsulating a hydrophobic core utilizing a hollow, hydrophobic interior structure as a carrier.	Durable storage and straightforward preparation without the need for specialized equipment.	Demands a same core diameter, leading to little product burden.	[34]

### 7.3. Physicochemical Processes

#### 7.3.1. Liposome Encapsulation

Liposomes are vesicular arrangements made of lipids stacked in bilayers or multilayers that may encapsulate hydrophilic as well as hydrophobic biologically active substances, such as cholesterol or phospholipids, in order to encapsulate bioactive peptides [75]. The polar head portions of the lipids orient both inside and outward, and each of these lipid‐based chambers is made up of many lipid bilayers. Their makeup offers a dependable microencapsulation technique for the distribution of medications and bioactive ingredients. Liposomes provide barriers that are impervious to intestinal bacteria, bile salts, digestive fluids, and the enzymes found in the gastrointestinal and mouth cavities. As a result, they maintain biologically active compounds and facilitate their effectiveness and absorption [76].

Liposomes are divided into four types regarding the content of the membrane and their size: unilamellar, multilamellar, oligolamellar, and multivesicular. The most commonly used liposomes are unilamellar because of their easy fabrication and membrane properties, while oligolamellar liposomes present manufacturing challenges because of the need for controlled processes, which has led to their limited use for encapsulating bioactive chemicals. Multivesicular liposomes have gained considerable prominence in drug delivery applications. Liposomes are divided into three sizes: small (ranges 15–500 nm), large (ranges 500–1000 nm), and colossal (ranges greater than 1 μm lengthwise) [77].

A variety of strategies are available for liposome production. The primary approaches include emulsion synthesis, micelle‐forming detergent processes, lipid film dehydration, and solvent injection procedures. In order to create multilamellar vesicles using superior encapsulation efficiency, liposome synthesis usually begins with a dehydrated lipid film, following which a water‐soluble form of the bioactive agent is added. However, liposomes have a significant distribution of particle size, which may impact the release rate. The surface charge, dimensions, components, physicochemical properties, and particle size distribution of liposomes determine how effective they are as carriers for bioactive substances [77].

Nanoliposomes offer an alternative that improves encapsulation stability, efficiency, and functionality while controlling the release rate of bioactive peptides, thereby significantly increasing peptide bioavailability along with optimizing the pharmacokinetic properties of liposomes. Liposomes are effective carriers for encapsulating bioactive peptides because the peptides have excellent integration with the liquid core, but the efficiency of encapsulation is often still insufficient due to the uncontrolled as well as spontaneous formulation of liposomes influenced by entropy, and the peptides quickly penetrate the liposomal fluid barrier, resulting in the uncontrolled release of biologically active compounds owing to their insufficient physical stability in environmental conditions [78].

Biologically active peptides derived from sources, such as peanuts [75] and beans, are stabilized via the creation of nanoliposomes. The effectiveness of biologically active peptide encapsulation by the formation of liposome is significantly lower than that of other encapsulation techniques because hydrophilic peptides via legumes inhibit them from engaging with the phospholipids that act as wall material, altering the structure of the lipid bilayer and leading to pore formation. Microfluidization is a technique used in liposome production to increase the encapsulation yield. According to Gong et al. [79], the application of homogenization pressure throughout microfluidization improves encapsulation efficacy and reduces particle size dispersion. Furthermore, other lipid molecules, such as 1,2‐dioleoyl‐3‐trimethylammonium propane, and 1‐palmitoyl‐2‐oleoyl‐sn‐glycero‐3‐phosphocholine [80], may be integrated to diminish the permeability of phospholipid membrane.

#### 7.3.2. Emulsification

Emulsification is referred to as “the procedure for dispersing a single liquid within an additional immiscible liquid.” The integration of a core material onto the initial liquid facilitates the encapsulation of bioactive substances [67]. There are two types of emulsions: water/oil as well as oil/water emulsions, such as water/oil/water, or as duel emulsions of oil/water/oil. Therefore, both hydrophilic and lipophilic active ingredients in food items can be delivered using emulsions. Based on the formula and processing circumstances, the particle size can be significantly smaller, although it typically ranges between 50 and 500 μm. Emulsification’s growth potential and versatility in using different coating materials are its advantages. Numerous more processes are involved, including oil separation and emulsification. Traditional emulsions can be replaced with multilayered emulsions. Several oil‐in‐water‐in‐oil (O1/W/O2) emulsions may be created using a hydrophilic emulsifier that stabilizes the O1/W interface as well as a hydrophobic emulsifier, which stabilizes the O2/W interface. Because of the encapsulated naturally active component in the internal oil phase, the characteristics of the two oil phases in these emulsions may be the same or different. Furthermore, ω‐3 oils have recently been encapsulated using multilayered emulsions [81].

#### 7.3.3. Ionic Gelation

Ionic gelation is one kind of physicochemical encapsulating technique. Because it eliminates the requirement for organic solvents and harsh conditions, this method has attracted a lot of interest lately due to its novelty and attractiveness [82]. Ionic interactions between oppositely charged substances, which might involve a pair of polymers or a single polymer and a divalent ion, are the basis of this technique. Creating a polymer in a liquid solution that reacts with a substance that has an opposing charge to produce an insoluble gel is the first step in the encapsulation process. A dispersion, solution, or emulsion is produced when the active ingredient is often added to the polymer solution. When a solution, dispersion, or emulsion is atomized onto a divalent ion combination while being constantly stirred, gel formation takes place.

The active element is uniformly encased in the polymer matrix by the spherical gel formations produced when the droplets react with an ionic solution. The two phases of the process are solidification and capsule creation. The first phase determines the particle size, whereas the second phase encourages interactions among the wall materials with divalent ions, leading to capsule hardening [83].

The interactions among the wall components and divalent ions determine how well peptides are encapsulated within ionic gelation. In order to create the network that wraps peptide molecules, divalent ions must interact with the polymers [84]. Maintaining an ideal equilibrium between the concentrations of these chemicals is so crucial. Because of a saturated condition of sodium alginate sites of binding by Ca^2+^ ions, increasing the quantity of calcium chloride in the solution (2%–3% w/v) lowered the encapsulation effectiveness of the protein hydrolysate via *Ziziphus jujube*. The efficacy of encapsulation is also impacted by the wall material mixture’s higher viscosity [85]. This causes the peptide to dissolve prematurely onto the aqueous medium prior to encapsulation, resulting in the development of big and irregular droplets where polymer network building and divalent ion generation occur at a reduced pace [86].

Ionic gelation has become an effective encapsulation method that preserves the biological activity of bioactive peptides derived from rice husks [84], soybean lupine [87], and *Z. jujube* seeds [85]. Only 65% of the encapsulated peptides had been released after 6 days, according to the in vitro diffusion profile of rice husk extract peptides at neutral pH. The strength of the ensuing gel and the interactions of intermolecular between the peptide and the hydroxyl groups of the material that forms the wall are the causes of this phenomenon [84]. Using modest quantities of wall materials that stay ungelded in the presence of ions that is divalent and help to decrease gel strength, such as xanthan gum, is an alternate method to increase the release percentage of peptides of the plant [88].

Despite its mild processing conditions, ionic gelation often produces highly porous structures that enable burst release, limiting its applicability in controlled‐release food systems [25]. Additionally, scale‐up remains challenging due to sensitivity to ion concentration and viscosity fluctuations, restricting its current use largely to laboratory‐scale applications. Quantitative analysis of published ionic gelation protocols reveals significant batch‐to‐batch variability: coefficient of variation (CV) values for particle size commonly range from 15% to 35%, and encapsulation efficiency CV values of 10%–25% are frequently reported, compared to < 10% CV for spray drying under controlled conditions. This variability stems from the rapid gelation kinetics and sensitivity to mixing conditions, droplet formation rate, hardening time, and ion diffusion rates parameters difficult to control precisely at large scale. Furthermore, the requirement for large volumes of cross‐linking solution (typically 10–50 fold excess relative to polymer solution) generates substantial liquid waste streams, raising both economic and environmental concerns. Current production capacities for ionic gelation remain limited to < 10 kg/hour even in optimized semicontinuous systems, compared to 100–1000 kg/hour for spray drying, fundamentally limiting its industrial applicability outside niche high‐value applications [38, 42, 43]. The principles, advantages, and disadvantages of physicochemical processes are summarized in Table 6.

**TABLE 6:** Technical principles, advantages, and disadvantages of physicochemical processes.

Encapsulation system	Principle	Advantages	Disadvantages	References
Liposome encapsulation	The wall material comprises a spherical or nearly spherical vesicle characterized by a biofilm structure, generally composed of more than one phospholipid bilayers or just thin layers.	Efficiently encapsulates molecules within a phospholipid bilayer, minimizing deterioration in harsh conditions.	Challenging extraction of organic solvents including synthetic surfactants, necessitating stringent storage conditions.	[89]
Emulsification	An emulsion in the oil–water system is created by including suitable surfactants through vigorous mixing, resulting in the formation of colloidal particles that enclose and safeguard the core substance.	Streamlined production method, enhanced digestibility, antimicrobial properties, and antioxidant effects.	Exhibits low physical rigidity and susceptibility to demulsification under severe conditions.	[90]
Ionic Gelation	Employs ionic interactions (e.g., alginate as well as calcium ions) to create gel‐like encapsulating structures.	Cost‐effective manufacturing technique with minimal temperature requirements.	The capsule generated is characterized by high porosity, resulting in intense bursts and challenges in regulation, commonly utilized in laboratory‐scale manufacturing.	[25]

## 8. Industrial‐Based Applications of Encapsulation in Food Industry

Encapsulation technology is extensively employed across several sectors, particularly in food industries, since it improves stability, enhances solubility, and optimizes the release characteristics of chemicals such as EOs, enzymes, antioxidants, and pharmaceuticals [37]. Applications for encapsulation methods are quite broad in many different sectors [91].

In the food sector, encapsulation serves multiple purposes, chief among them the enhancement of ingredient stability and bioavailability, particularly in functional food applications. Beneficial meals are distributed and encapsulated using a variety of targeted delivery techniques, including nanoemulsions, emulsion bilayers, and surfactant micelles [92]. The food business uses functional additives to improve flavor, texture, color, and extend product shelf life [93]. Furthermore, substances with practical health advantages, such as probiotics and antioxidants, are widely valued. However, the majority of these substances are unstable and readily broken down by environmental conditions [94]. Thus, it is essential to synthesize highly stable bioactive molecules. One method used to solve these problems is encapsulation. In these years, a lot of research has focused on the production of high‐yield microcapsules including their use in the food industry. The section examines the applications in different food industries of encapsulation.

Although successful industrial applications of encapsulation have been reported across beverages, dairy, bakery, and meat products, most implementations rely on incremental improvements rather than disruptive innovation. Encapsulation is frequently used to protect existing ingredients instead of enabling novel formulations or processing paradigms, indicating that its transformative potential in the food industry remains underexploited [37, 95]. A critical analysis of commercial encapsulation applications reveals that approximately 70%–80% of industrial implementations utilize spray‐dried microcapsules with conventional wall materials (MD, GA, modified starches), primarily for flavor protection and powdered oil production. Only 10%–15% of applications employ more sophisticated techniques (coacervation, liposomal systems, multilayer emulsions), and these are concentrated in premium product categories where higher costs can be justified. This conservative industrial approach reflects risk‐averse decision‐making prioritizing proven technologies over potentially superior but less‐established methods [45, 57]. The gap between academic innovation (where > 50% of publications focus on novel techniques and materials) and industrial adoption (where > 80% use established spray drying) exemplifies the disconnect this review addresses.

**FIGURE 3:**
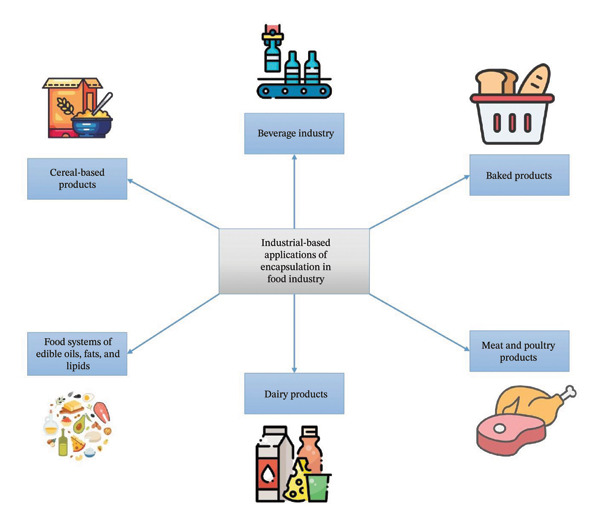
Industrial‐based applications of encapsulation.

Furthermore, sustainability metrics for industrial encapsulation processes remain poorly documented and rarely guide technology selection. Recent life‐cycle assessment (LCA) studies comparing encapsulation techniques for vitamin E delivery in dairy products revealed substantial differences in the environmental impact: spray drying exhibited energy consumption of 6.2 MJ/kg product and carbon footprint of 1.1 kg CO_2_eq/kg, compared to freeze drying (28.4 MJ/kg, 4.8 kg CO_2_eq/kg) and coacervation (8.7 MJ/kg, 1.6 kg CO_2_eq/kg). Despite inferior encapsulation efficiency (65%–75% vs. 85%–95% for coacervation), spray drying’s superior sustainability profile justified its selection for this application [46, 58, 83, 85]. Such integrated decision‐making considering both performance and sustainability represents the approach advocated by our proposed framework but remains absent from most industrial technology selection processes. Some industrial‐based applications of encapsulation in the food industry are illustrated in Figure 3.

### 8.1. Beverage Industries

Prasetyaningrum et al. [96] assessed the stability of anthocyanin, which is encapsulated in several carrier agents inside an isotonic soft drink platform. Anthocyanins contain water‐soluble pigments derived from plants. These pigments are typically employed as colorants within food and beverages due to their potent coloring ability, low toxicity, and great solubility in water. Furthermore, numerous studies have demonstrated the strong anticarcinogenic and antioxidant qualities of anthocyanins. The anthocyanins are unstable pigments that can decompose into colorless materials under a number of circumstances, including light, temperature, oxygen, pH, and the food matrix. The microencapsulation procedure has therefore been utilized to improve the stability of these chemicals. The study encapsulated anthocyanins that were isolated from the Cabernet Sauvignon grapes using the spray‐drying procedure. The resulting microcapsules were spherical in shape and had consistent particle sizes. Furthermore, the stability of pigments containing anthocyanin was enhanced by the mixture of GA and MD [97].

Aditya et al. [98] produced curcumin and catechin microcapsules using W/O/W emulsions. Preventing the degradation of curcumin and catechin in beverage systems was the aim of this investigation. Its biological efficiency is increased when curcumin and catechin are combined. Since these two compounds are strong bioactive agents that can prevent a variety of diseases, including cancer, obesity, infections, and heart disease, the food industry uses them to make functional food and drink products. Curcumin and catechin, however, are unstable components. When exposed to alkaline pH, oxygen, and high temperatures, they readily break down [99].

Indiarto et al. [97] used the spray‐drying procedure to encapsulate the oil of lemon using MD. The aroma of lemon oil is powerful and invigorating. Consequently, it is mostly used as a flavoring additive in drinks and culinary applications. Oxidation during storage is impacted by this oil’s large amounts of unsaturated and oxygen‐functionalized components. Consequently, the microencapsulation technology has now been employed to resolve this issue. The stability of the isolates was assessed for sensory properties of instant iced tea premix at different storage temperatures (4°C, 28°C, and 45°C). Encapsulated lemon oil exhibited a taste profile and favorable odor, with no alteration in appearance during the storage duration under all circumstances. The findings demonstrated that encapsulated lemon oil is capable of being preserved for a duration of 6 months.

Despite these technical successes, commercial implementation faces scalability challenges rarely discussed in research publications. Industrial beverage production lines operate at speeds of 200–1500 bottles/minute, requiring instantaneous dissolution and dispersion of encapsulated ingredients without agglomeration, sedimentation, or creaming. Spray‐dried microcapsules sized 10–50 μm generally meet these requirements, but smaller nanoparticles (< 1 μm) often exhibit problematic aggregation in high‐ionic‐strength beverage matrices, and larger particles (> 100 μm) may cause mouthfeel issues or sedimentation. Furthermore, beverage pH (typically 2.5–4.5 for soft drinks) and pasteurization treatments (72°C–95°C for 15–30 s) place additional stability demands on encapsulated ingredients that bench‐scale studies often fail to replicate [59, 60]. Economic analysis indicates that encapsulated ingredients for beverages must typically add < $0.05 per liter to product cost to be commercially viable in mainstream products, constraining the use of expensive techniques or materials to premium categories.

### 8.2. Baked Products

Using a modified starch to provide an encapsulating environment, Rocha et al. [100] made lycopene microcapsules by using the spray‐drying procedure. Applying microcapsules to cake allowed for the evaluation of their efficacy. One kind of carotenoid found in various vegetables and fruits is lycopene. A common use for it is as a red coloring for food. However, due to its massive conjugated double‐bonded structure, lycopene is rapidly damaged by oxidation during storage. According to this study, microencapsulation will increase lycopene’s stability. The outcomes showed that the microcapsule‐made cake was more pigmented than the conventional cake. In order to enhance oxidative stability and transform fat into fixed powder for the manufacturing of short dough cookies, Peanparkdee et al. [37] enclosed vegetable oil. At now, the majority of components utilized in the creation of commercial biscuits are in a desiccated state. Nevertheless, the fat component must be included as either a liquid (oil) or solid (fat), necessitating an extra manual procedure. According to research, whey protein concentrate (WPC) with 5% protein for the agent of encapsulation and microencapsulated vegetable fat generated at low synthesis pressure are suitable for creating biscuits with acceptable properties. Therefore, when making commercial biscuits, high‐fat powders that have been microencapsulated can be utilized in place of fat or oil.

Baking applications present particularly demanding conditions for encapsulated bioactives: oven temperatures of 150°C–220°C, low‐moisture environments (water activity 0.3–0.6 in finished products), and extended shelf life requirements (3–12 months under ambient conditions). These conditions necessitate wall materials with exceptional thermal stability and low moisture permeability. Industrial‐scale bakery operations also require encapsulated ingredients compatible with high‐speed mixing (1000–3000 rpm), dough sheeting, and cutting processes without premature release or particle breakage [61, 62]. Cost constraints are particularly stringent in bakery applications, where profit margins are typically low; encapsulated ingredients must usually add < $0.10–0.30 per kg to dough formulation cost, strongly favoring spray‐dried microcapsules over more expensive techniques. A notable success story is the commercial‐scale microencapsulation of omega‐3 oils for bread enrichment, achieving shelf life stability of 6–9 months without oxidative off‐flavors, but requiring careful optimization of particle size (20–40 μm), wall composition (protein‐carbohydrate blends), and antioxidant coencapsulation to meet both performance and cost targets [63].

### 8.3. Poultry and Meat Products

Peanparkdee et al. [37] investigated the consolidation of probiotics into dried air‐fermented sausages to enhance nutritional value. Numerous research studies have indicated that probiotic microbes exhibit inadequate survival rates in fermented meals. The microencapsulation technology was utilized to prolong the viability of bacterial cells through encasing them within a membrane of polymer that is either protective or just a matrix. The results demonstrated that microencapsulated *Lactobacillus reuteri* is suitable for dry fermented foods, since it maintains survival of the cell after drying while preserving the sensory qualities of the products.

In order to enhance frozen chicken nuggets, Jiménez‐Martín et al. [81] used microcapsules containing fatty acids of omega‐3 derived from fish oil. They also examined the impact of frozen storage duration on the product’s oxidative stability and sensory qualities in comparison to that of bulk fish oil incorporation. The overall taste of chicken nuggets enhanced with omega‐3 fatty acids was unaffected by the freezing time. By extending the oxidative shelf life and maintaining sensory quality attributes, omega‐3 fatty acid microencapsulation from the oil of fish may enhance prefried frozen beef products.

The impact of ascorbic acid that is encapsulated on the physicochemical as well as sensory persistence of chicken frankfurters was assessed by Comunian et al. [101]. Vegetables and fruits are a natural source of ascorbic acid, an antioxidant. However, it is quite unstable. A number of things, such as heat, light, elevated level of oxygen, and high moisture content, may easily harm it. In place of sodium erythorbate, ascorbic acid is frequently used in frankfurters.

Meat product applications face unique challenges combining low pH (5.0–6.0), high salt content (1.5%–3.5%), lipid‐rich matrices prone to oxidation, and thermal processing (cooking to internal temperatures of 70°C–75°C). Encapsulated probiotics for fermented meats must survive not only processing but also the acidic, high‐salt fermentation environment (pH 4.5%–5.5%, 2%–4% salt) for 24–72 h. Industrial success requires achieving > 10^6^ CFU/g viable cells in the final product after processing and throughout shelf life—a target met by < 40% of encapsulation systems tested in research studies when subjected to realistic processing conditions [64, 65]. For omega‐3 enrichment of meat products, oxidative stability during frozen storage (−18°C for 6–12 months) is critical; successful commercial formulations typically achieve peroxide values < 10 meq/kg and TBARS < 1.0 mg MDA/kg at end of shelf life, requiring sophisticated wall materials (often protein–polysaccharide–antioxidant combinations) and careful control of residual oxygen in packaging. The additional cost of encapsulated omega‐3 oils ($15–35/kg vs. $3–8/kg for bulk fish oil) must be justified by measurable shelf life extension or superior sensory properties to achieve market acceptance [66].

### 8.4. Dairy Products

Using a variety of wall materials, Jiménez‐Martín et al. [81] created Flavourzyme microcapsules for use in the manufacturing of cheese. For low‐moisture cheeses like cheddar to acquire the required flavor, texture, taste, and fragrance qualities, they must be aged for a long time. Due to the sluggish rate of conventional cheese maturing, exogenous enzymes are used to accelerate the process. Nonetheless, the direct incorporation of enzymes leads to enzyme loss, inadequate enzyme dispersion, diminished production, and substandard cheese quality. Microcapsules incorporated into milk during cheese production facilitated effective dissociation of Flavourzyme.

Champagne et al. [102] assessed how the spray‐drying method of microencapsulation affected the probiotic bacteria’s durability in ice cream. Probiotic micro‐organisms have been used in the creation of several functional ice creams in recent years. However, handling and storage circumstances have an impact on the life of probiotic micro‐organisms. In order to increase the number of the lifespan of probiotic bacteria, microencapsulation has been used. When compared to the nonencapsulated culture, the results revealed that the usage of encapsulated beneficial bacteria had excessive survival rates.

Dairy applications represent the most mature and commercially successful category for probiotic encapsulation, with numerous products achieving global market penetration. However, this success reflects specific favorable conditions: neutral to slightly acidic pH (4.0–6.8), refrigerated storage (2°C–8°C), and relatively short shelf life (14–60 days) compared to ambient‐stable products. The regulatory landscape for probiotics in dairy is also well‐established, with species like *L. acidophilus*, *Bifidobacterium lactis*, and *L. rhamnosus* GG having extensive safety documentation and permitted use in most markets [67, 68]. Industrial‐scale probiotic encapsulation for dairy products predominantly uses extrusion‐dripping into calcium chloride solution (for alginate beads) or spray drying with protective matrices (skim milk powder, whey protein, prebiotic fibers), achieving production capacities of 50–200 kg/hour and costs of $8–18/kg encapsulated product economically viable for premium yogurts and probiotic drinks but challenging for mainstream products. A critical success factor has been the development of industry‐wide standards for probiotic viability claims (typically 10^6^–10^9^ CFU per serving at end of shelf life) and standardized testing protocols, facilitating regulatory approval and consumer trust [69].

### 8.5. Cereal‐Based Products and Functional Snack Products

One of the most rapidly developing branches of the food industry with encapsulation technology playing central industrial role consists of cereal‐based foods and ready‐to‐eat functional snacks. High‐temperature processing, shear stress, and prolonged storage conditions are widely used in breakfast cereals, extruded snacks, nutrition bars, and fortified flours and have the potential to seriously degrade heat‐sensitive bioactive compounds including vitamins, polyphenols, omega‐3 fatty acids, and minerals. Encapsulation has thus been embraced to safeguard such functional ingredients and make them stable through the process of extrusion, baking, and storing [103].

Microencapsulation of iron, zinc, folic acid, and vitamin A usage has been of wide use in fortified cereal products to avoid unwanted interactions with cereal components, such as phytic acid, which may lower the bioavailability of the micronutrients. Encapsulated minerals have enhanced dispersibility and alleviated sensory flaws such as metallic taste, discoloration, and off‐flavors in cereal matrices. It has been demonstrated that lipid or carbohydrate‐based encapsulated iron has a significant positive effect on iron retention and bioaccessibility when added to fortified wheat flour and breakfast cereals in comparison with unencapsulated iron [104].

Encapsulation of polyphenols and plant extracts improves the antioxidant stability of functional snack bars and extruded products and prevents degradation of these products during extrusion at high temperatures. Omega‐3 fatty acids have also been easily added to cereal snacks, as either spray‐dried or lipid‐encapsulated to preserve relative oxidative stability and sensory properties. The implementation of encapsulated bioactives in cereal systems by the industries facilitates a manufacturer to address the nutritional labeling demands without affecting the quality, shelf stability, and consumer acceptability of the products [105].

Cereal and snack applications exemplify the scalability–performance trade‐off central to this review’s thesis. Extrusion processing is the dominant technology for breakfast cereals, snack bars, and puffed snacks subjects ingredients to extreme conditions: temperatures of 120°C–180°C, pressures of 25–100 bar, shear rates of 100–1000 s^−1^, and residence times of 30–180 s. These conditions destroy > 90% of unprotected vitamins and probiotics and cause > 70% oxidation of unencapsulated omega‐3 oils [70, 71]. Effective encapsulation can reduce these losses to 10%–30%, but only if capsules withstand both the mechanical shear (which can fracture brittle particles) and thermal stress without releasing contents prematurely. Industrial success requires particles sized 50–300 μm (smaller particles degrade during extrusion; larger particles cause textural defects), moisture content < 5% (to prevent steam‐induced bursting), and glass transition temperatures > 100°C (to maintain structural integrity). These specifications effectively limit viable techniques to spray drying with high‐Tg wall materials (modified starches, MDs, whey protein isolate) or specialized lipid‐based encapsulation systems, despite the availability of numerous alternative techniques with superior laboratory performance [72, 73].

The mineral fortification of cereals presents additional regulatory complexity. Iron fortification is mandated in many countries (e.g., wheat flour fortification at 30–60 mg Fe/kg in > 80 nations), but encapsulated iron must meet bioavailability requirements (typically > 30% relative bioavailability vs. ferrous sulfate reference) while avoiding sensory degradation (color changes, rancidity) during 9–18‐month ambient storage [74]. This has driven development of specialized encapsulated iron systems (e.g., ferrous fumarate in lipid matrices, NaFeEDTA in starch‐protein coatings) that balance bioavailability, stability, and cost ($3–8/kg for encapsulated premixes vs. $2–4/kg for nonencapsulated salts). However, economic constraints in cereal fortification programs particularly for humanitarian applications in developing countries often prioritize cost over optimal performance, illustrating how decision‐making criteria shift across market segments [34, 35].

### 8.6. Food Systems of Edible Oils, Fats, and Lipids

The encapsulation technology has become of great industrial concern in stabilization and functionalization of food systems based on oils and lipids and their derivatives. PUFAs, especially omega‐3 and omega‐6 fatty acids, are very susceptible to oxidative degradation by oxygen, light, and heat. This can only be incorporated directly in foods such as spreads, dressings, margarine, powdered fats, and instant foods in a limited manner. An efficient approach for preserving these lipids and prolonging their shelf life is encapsulation [106].

Conversion of liquid oils into stable powdered forms that are easily incorporated into the formulations of dry food is commonly done by spray drying, complex coacervation, and liposomal encapsulation. Fish oil and flax oil in encapsulated form have been effectively used in infant formulas, powdered milk substitutes, instant soups, and bakery premixes. Encapsulation has a tremendous impact on inhibiting lipid oxidation, covering fishy off‐flavors and enhancing consumer acceptance with no nutritional value change [107].

Structured emulsions with the feel of conventional fats are also produced by the use of encapsulated lipid droplets in industrial fat‐replacement systems to reduce the overall fat content. Also, antioxidant agents including tocopherols, rosemary extract, and phenolic acids are often coencapsulated with oils to further improve the oxidative stability. Lipid‐based food systems using encapsulated oils help in producing healthier foods that have long shelf life, better sensory properties, and better nutritional functionality [108].

Omega‐3 encapsulation represents perhaps the most commercially significant and challenging application of food encapsulation technology. The global market for omega‐3 ingredients exceeded $2.4 billion in 2023, with encapsulated forms commanding premium pricing ($18–45/kg vs. $8–15/kg for bulk oils) due to superior stability and versatility [75]. However, achieving commercial success requires meeting stringent oxidative stability targets: peroxide value < 5 meq/kg and anisidine value < 20 at the end of shelf life (typically 18–24 months), while maintaining the EPA + DHA content within ±10% of label claim. These targets necessitate multibarrier protection strategies: oxygen‐impermeable wall materials, antioxidant incorporation (typically 200–500 ppm mixed tocopherols, 50–200 ppm rosemary extract), low‐oxygen processing environments (< 2% O_2_), and moisture‐barrier packaging [76, 77].

Industrial omega‐3 encapsulation predominantly employs spray drying (65%–70% of market), with coacervation (15%–20%), lipid matrices (10%–12%), and other techniques (< 5%) comprising the remainder. This distribution reflects the cost–performance optimization: Spray drying achieves adequate oxidative stability (typically 12–18‐month shelf life) at competitive costs ($2.50–4.50/kg processing cost), while coacervation offers superior protection (18–30‐month shelf life) but at 40%–80% higher processing costs. The choice between techniques depends on product positioning: mainstream supplements and fortified foods use spray‐dried omega‐3, while premium infant formulas and medical nutrition products justify coacervation economics [78]. This market segmentation by technique, based on value–price–performance analysis, exemplifies the decision framework approach advocated in this review but rarely articulated in academic literature. The impact of encapsulation on stability, bioavailability, and food application performance is summarized in Table 7.

**TABLE 7:** Impact of encapsulation on stability, bioavailability, and food application performance: performance metrics and industrial feasibility.

Encapsulated bioactive	Encapsulation approach	Improvement achieved	Food application	Performance outcome	Industrial implementation status	Estimated cost impact (USD/kg product)	References
Essential oils (lemon, clove)	Spray drying, ionic gelation	Reduced volatility (60%–80% retention vs. < 20% free), enhanced antimicrobial activity	Beverages, bakery, edible films	Extended shelf life (+30–50%), maintained aroma	Commercial (spray drying); laboratory only (ionic gelation)	+0.80–2.50 (SD); +3.50–8.00 (IG)	[97, 109]
Polyphenols/carotenoids	Electrospinning, nanoemulsion	Increased water solubility (5–20 ×), antioxidant stability (70%–90% retention vs. 20%–40% free)	Yogurt, beverages	Improved color stability (+50–120 days), antioxidant retention	Pilot scale only (electrospinning); commercial limited (nanoemulsion)	+5.00–15.00 (ES); +2.00–6.00 (NE)	[53, 110]
Probiotics	Extrusion gelation, spray drying	Improved GI survival (10^2^–10^3^ fold vs. free cells), storage stability	Dairy, meat products	Higher viable counts (> 10^6^ CFU/g vs. < 10^4^ free)	Commercial (both techniques, with spray drying dominant)	+1.50–4.00 (SD); +2.50–6.00 (EG)	[59, 102]
Vitamins (A, C, E)	Emulsification, liposomal systems	Enhanced thermal stability (70%–85% retention vs. 30%–60% free at 100°C), controlled release	Functional foods, fortified cereals	Improved bioavailability (+40–120%), retention (+50–200%)	Commercial (emulsification for A, E); Pilot/limited (liposomal)	+0.60–2.00 (emulsion); +4.00–12.00 (liposomal)	[42, 43]
Omega‐3 oils	Spray drying, coacervation, lipid matrices	Oxidative stability (PV < 5 meq/kg for 12–24 months vs. < 3 months free), odor masking	Bakery, dairy, supplements, infant formula	Extended shelf life, consumer acceptance	Commercial (all three techniques at industrial scale)	+2.50–4.50 (SD); +4.50–8.00 (coac.); +3.50–6.00 (lipid)	[106–108]

*Note:* Cost impacts represent the incremental production costs per kg of final food product, estimated from industrial case studies and supplier data (2023–2024). Implementation status categorizes commercial adoption: “Commercial” indicates > 10 industrial‐scale implementations globally; “pilot/limited” indicates 1–10 pilot or niche commercial applications; and “laboratory only” indicates no industrial‐scale implementation despite published research. ES = electrospinning; NE = nanoemulsion; Coac. = coacervation. These data illustrate the poor correlation between research publication volume (> 200 papers on electrospinning and ionic gelation for food applications in 2020–2024) and industrial adoption (near‐zero commercial implementation), highlighting the technology–implementation gap that motivates this review [79, 80].

Abbreviations: EG, extrusion gelation; IG, ionic gelation; SD, spray drying.

## 9. Sustainability and LCA of Encapsulation Technologies

A critical dimension absent from most encapsulation research yet increasingly decisive for industrial technology selection is environmental sustainability. As food manufacturers face growing regulatory pressure (EU Green Deal, corporate net‐zero commitments) and consumer demand for sustainable products, the environmental footprint of encapsulation processes becomes a key decision criterion alongside technical performance and economics [81, 82]. A comparative LCA of major encapsulation techniques is presented in Table 8.

**TABLE 8:** Comparative life‐cycle assessment of major encapsulation techniques for bioactive delivery.

Technique	Energy consumption (MJ/kg product)	Water usage (L/kg product)	GHG emissions (kg CO_2_eq/kg product)	Organic solvent use (kg/kg product)	Waste generation (kg/kg product)	Overall sustainability score (1–10)	References
Spray drying	6.2–8.5	8–15	1.1–1.4	0 (aqueous)	0.15–0.30	7.5	[46, 83]
Freeze drying	28.4–35.2	12–20	4.8–5.9	0 (aqueous)	0.10–0.20	3.5	[46, 84]
Coacervation	8.7–12.4	45–120	1.6–2.3	0–0.05	5.5–12.0 (crosslink solution waste)	5.0	[58, 85]
Emulsification + spray drying	7.8–10.2	10–18	1.3–1.7	0–0.02	0.20–0.40	6.5	[86]
Ionic gelation	4.2–6.8	85–200	0.9–1.5	0 (aqueous)	8.5–20.0 (gelation solution waste)	4.0	[43, 87]
Liposomal (solvent method)	15.2–22.5	25–40	2.8–4.2	0.8–2.5 (chloroform, ethanol)	0.8–3.0 (solvent waste)	3.0	[88]
Electrospinning	18.5–28.0	5–12	3.2–4.8	0.5–1.8 (various)	0.15–0.40	3.5	[89]

*Note:* Data represent the ranges from published LCA studies and industrial process documentation. Sustainability score (1–10, higher is better) integrates energy, water, emissions, solvent use, and waste metrics with equal weighting; scores > 7 indicate “Sustainable,” 5–7 “Moderate Impact,” < 5 “High Environmental Impact.” These data reveal that spray drying despite moderate energy consumption achieves the best overall sustainability profile due to zero solvent use, minimal waste generation, and high throughput, enabling economies of scale. Conversely, techniques like freeze drying and electrospinning, often promoted for superior product quality, exhibit environmental footprints 3–5 × higher than spray drying, raising questions about their viability in sustainability‐conscious markets [81, 90].

Critical analysis of these LCA data reveals several insights:

1. Energy–Performance Trade‐off: Several techniques provide outstanding encasement efficiency (such as freeze drying ∼ 85–95% efficient) and stability (such as electrospinning with controlled nanofiber morphology); however, they typically consume three to five times as much energy to produce compared to the spray‐drying method (65%–80% efficient and stable but not as effective). For many applications, the improved performance gained by using these various processes does not justify the sustainability and/or cost impacts caused by significantly increasing their associated energy consumption [83, 84].
2. Water and Waste Burden: The production of large amounts of liquid waste (gelation solutions, washing water, excess cross‐linker) from both ionic gelation and coacervation techniques and the requirements of pretreatment of these wastes before final discharge are a major impact area from both a water and waste consideration. The costs associated with wastewater treatment in industrial operations that utilize these processes can be in the range of $0.50–$2.00 per cubic meter, while producing a total cost of between $4.25 and $40.00 per kilogram of product produced; in many cases, the wastewater treatment costs exceed the value of the encapsulated ingredient [85, 87].
3. Solvent Recovery Economics: An alternative is to recover solvents used during liposomal and emulsification techniques that have utilized organic solvents (chloroform, ethanol, and hexane). Recovery efficiencies can be achieved in the range of 85%–95% using distillation; however, many facilities do not have the capital cost ($50,000–500,000) or energy costs (3–8 MJ/kg recovered) to recover solvent, thus making these techniques unviable for small‐ to medium‐scale processes (< 1000 kg/day) except for high‐value products [88, 91].
4. Renewable Energy Integration: Several industrial encapsulation facilities have integrated renewable energy (solar, wind) to reduce carbon footprints, achieving 40%–60% reductions in GHG emissions for spray drying (from 1.1 to 1.4 to 0.5–0.8 kg CO_2_eq/kg). However, high‐temperature processes like freeze drying remain challenging to decarbonize due to their > 200°C heat requirements [92].

The emerging regulatory landscape increasingly mandates environmental disclosures. The EU’s proposed Ecodesign for Sustainable Products Regulation (2024) will require digital product passports documenting environmental footprints for food ingredients, including encapsulated bioactives [93]. This regulatory shift will fundamentally alter technology selection criteria, prioritizing techniques with favorable LCA profiles even if technical performance is marginally inferior, a paradigm shift from current practice.

## 10. Future Prospects and Research Priorities

Future encapsulation research should transition from maximizing encapsulation efficiency toward optimizing system‐level performance under realistic industrial constraints, ensuring that technological innovation aligns with sustainability and regulatory feasibility. Over the last ten years, there has been an increased surge in research and commercial opportunities in the use of biologically active compounds as antibacterial and antioxidants in the food industry. The increasing demand of consumers in safe, minimally processed food with longer shelf life and sensory quality preservation has increased the rate of uptake of innovative food preservation approaches. Most notable among them are encapsulation technologies, which have received specific focus as useful methods to preserve delicate bioactive compounds and allow them to be released into food systems controlled. An assortment of food products consisting of meat, dairy products, eggs, fruit juices, spreads, bakery, yogurt‐based beverages, and functional snacks is being fortified with bioactive components actually vitamins, probiotics, omega‐3 fatty acids, and phytochemicals in the growing proportion to boost nutritional worth and functionality [111].

The most popular method of producing microencapsulations, wherein the technique is commercialized in the food industry, is spray drying, owing to its scalability, cost‐effective applications, and ease of operations. Other encapsulation methods, such as complex coacervation, electrospraying, and inclusion complex formation, are however increasingly being considered by research attention due to their aptitude in enhancing encapsulation efficiency and bioactive stability. Complex coacervation has shown promising results with regard to the high encapsulation yield and sustainability; however, its usage in the industrial field is limited by scarcity and high cost of wall materials used. Gelatin mixed with GA is the most widely used in the present coacervation system. Recent research reveals that associations of plant proteins with polysaccharides are capable of producing complex coacervates with benefits such as reduced cost, increased availability, and environmental sustainability, and thus, their further study in terms of food application is beneficial [36]. It is worth noting that microcapsules of EOs synthesized under complex coacervation have been proven to be effective at inhibiting the growth of microbes in both fresh and processed foods such as fruits, milk, juices, vegetables, and bakery products.

Other emerging technologies in the controlled delivery of hydrophobic food bioactives include inclusion complex technology. The host–guest complexes in these systems are due to the noncovalent interactions between the host and guest, which enable the molecular capture of hydrophobic molecules within the host cave during aqueous solution conditions. This method enhances the solubility and the stability without chemically altering the encapsulated compound. Inclusion complexes with cyclodextrins have been reported to increase the stability, antioxidant properties, and antimicrobial intended properties of food‐grade active compounds, and it, therefore, has proven to be the most suitable inclusion complex in food preservation and functional food formulations [34].

Electrospraying is also a latest form of encapsulation method that has application in functional foods and food packaging. This is a technique that allows the introduction of antibacterial and antioxidant compounds into fine particles with high surface modes. Nevertheless, natural polymers that are most frequently utilized in the electrospraying of Ch, zein, and gelatin are rather costly and can also confer unwanted sensorial attributes. Moreover, the low production throughput of electrospraying is characteristic of this process; thus, it is one of the main reasons that limit the application of this technique at the industrial level, which is why the research on alternative, cost‐effective, and water‐soluble as well as sensory‐neutral biopolymers that are applicable to food systems is necessary [112].

Recent changes, like the production of nonbiodegradable synthetic preservatives to biodegradable ones, have increased the need to seek antimicrobial food packaging. Ch, cellulose, and zein biopolymers have demonstrated significant promise when used together with EOs, plant extracts, bacteriocins/bacteriophages, or plant extracts to a large degree in prolonging shelf life and improving food safety [51]. But the volatility and sensitivity of EOs to light and heat make them rather unstable and ineffective. Therefore, future studies must consider the enhanced methods of stabilization such as encapsulation and surface‐functionalized nanoparticles to enhance the stability and release of antimicrobial agents in the food packaging environment [113].

Even though most studies on the encapsulation of compounds have been conducted in vitro and in vivo, more detailed studies on interactions between encapsulated compounds and food‐matrix components are needed. The next type of research to focus on should be the analysis of the release kinetics, efficacy of the antimicrobials, and sensory effects in real food systems. Increasing oxidative stability of lipid‐containing foods, increasing sensory qualities and texture of functional foods, and effectively preventing microbial growth but not consumer acceptance are all research priority areas that would lead to successful commercialization of encapsulated bioactive compounds in the food industry [111].

Beyond these established research directions, several critical priorities emerge from this review’s analysis of the technology‐implementation gap:

### 10.1. Standardization and Performance Benchmarking

The lack of standardized testing procedures to measure the performance of encapsulation in the presence of industrially relevant conditions is a major ailment to technology translation. The current literature presents the encapsulation efficiency measurements in a variety of analysis tools (surface oil determination, extraction‐based quantification, indirect calculation), and interstudy comparison becomes invalid. The recommendations of the future research are to follow standardized methods that evaluate (1) encapsulation efficacy with validated extraction techniques (e.g., the Folch method of lipophilic cores, aqueous extraction of hydrophilic cores, extraction with triplicate determination and RSD under 5%), (2) stability in accelerated shelf life conditions simulating actual product storage (temperature, water activity, oxygen exposure, light), and (3) sensory effects of incorporation into target food matrices at target levels of use. Moreover, establishing performance standards for each application type (e.g., vitamin E in baked goods should retain more than 70% of its activity during processing at 180°C for 20 min and during storage at 25°C for 9 months) would enable comparative benchmarking of encapsulation methods and support informed technology selection. The development and validation of such protocols could be performed through industrial‐academic consortia on par with those that have developed probiotic viability criteria [97].

### 10.2. Machine Learning and AI‐Guided Formulation Optimization

The geometry of encapsulation systems (comprising multi‐ingredient collections of core, wall materials, coencapsulants, antioxidants), process conditions (temperature, pressure, flow rates, atomization conditions), and performance parameters (efficiency, stability, release kinetics) is too hard to be thoroughly investigated using traditional one‐factor‐at‐a‐time experimentation. Potential ways to speed up formulation optimization via machine learning techniques, especially artificial neural networks and evolutionary algorithms, are the possibility of finding complex nonlinear relationships between formulation variables and the performance outcomes [98, 99].

Recent uses have been made and proven successful: neural network models using 180 spray‐dried formulations identified encapsulation efficiency (*R* ^2^ = 0.89) and oxidative stability (*R* ^2^ = 0.84) of novel combinations of wall materials and exploited it to reduce experiments by more than 60% [100]. The further development of these methods to incorporate sustainability indicators (energy usage, waste production) and cost functions and performance together would allow holistic optimization in accordance with the decision framework suggested in this review. Nevertheless, these applications need big and quality‐rich datasets, which is not available in the existing literature that underscores the necessity of standardized data reporting and open‐access databases [101].

### 10.3. Circular Economy and Biorefinery Integration

Sustainable encapsulation should not be limited to work efficiency but should be able to look at the sourcing of raw materials and disposal at the end of the work. Food processing side‐streams and agricultural residues as the sources of wall materials have two advantages: utilizing the waste and saving costs. It has recently been shown to be possible to capture encapsulating agents using okara (soy processing residue), brewer’s spent grain proteins, fruit and vegetable peels (pectin, cellulose nanofibers), and seafood processing waste (Ch) [102–104]. Nevertheless, such materials are commonly found to be varied in appearance and behavior with respect to materials batch to batch and need purification and characterization principles before finding use in industrial sectors.

Use of encapsulation production in areas of biorefinery that converts biomass into various products of value may enhance economics due to shared infrastructure and use of stream of waste. Speaking of an example, a soy biorefinery that will produce protein isolates, oils, and oligosaccharides may make use of fractionated proteins as encapsulation wall material, cutting the costs of the process by half to two‐thirds of what the commercial protein isolates would cost [105]. These combined strategies demand cross‐sector cooperation among food processors, ingredient suppliers, and technology providers—organizational designs that are seldom considered in the scholarly literature but are vital in the industrial practice [106].

### 10.4. Regulatory Science and Proactive Safety Assessment

Regulatory uncertainty, particularly for nanotechnology‐based encapsulation systems, remains a major barrier to commercialization. Particles < 100 nm may trigger novel food or nanomaterial regulations requiring extensive toxicological data (90‐day oral toxicity, genotoxicity, reproductive toxicity studies) at costs of $200,000–800,000 and timelines of 2–4 years [107, 108]. This regulatory burden disproportionately affects small and medium enterprises lacking resources for comprehensive safety dossiers.

Proactive regulatory science research should focus on (1) developing predictive structure–activity relationships linking particle size, surface chemistry, and wall materials to bioavailability and potential toxicity, enabling risk assessment without exhaustive testing for each formulation, (2) establishing clear size and material thresholds distinguishing “conventional” from “novel” encapsulation systems requiring enhanced safety evaluation, (3) harmonizing regulations across jurisdictions (currently, a nanoencapsulated ingredient approved in the United States may face different requirements in EU, China, and Brazil), and (4) creating transparent, publicly accessible guidance for industry on safety data requirements and approval pathways [109, 110].

Notable progress includes the EFSA’s guidance on the risk assessment of nanomaterials in food and feed (updated 2021) and FDA’s draft guidance on nanotechnology (2022), but significant ambiguities persist, particularly regarding functional ingredients versus food‐contact materials and the applicability of “GRAS” status to nanoformulations of approved substances [111, 112].

### 10.5. Consumer Perception and Transparency

The information about consumer acceptance of encapsulated ingredients is not intensively explored even though it is the central requirement to succeed in the market. In surveys, the proportion suggesting that 40–60 percent of consumers doubt nanoparticles in foods or even microencapsulation in foods relates these words to artificial or excessively processed foods [113]. On the other hand, functional benefits (e.g., “protected probiotics that mitigate digestion”) and sustainability messaging (e.g., “cut food waste by increasing shelf life”) lead to a positive reaction in 65%–75% of consumers [114].

Future studies must examine (1) the best labeling and marketing strategies that convey the benefits without arousing negative perceptions, (2) readiness‐to‐pay premia above null rates (along demographic sectors and product lines) to the products with encapsulated ingredients, and (3) transparency (i.e., QR codes linking to the detailed information about the ingredients) that fosters trust. This kind of research in consumer science combined with technical development guarantees innovations keep in line with the market demand as opposed to innovations that produce technically outstanding products that are rejected by the consumer [115, 116].

## 11. Emerging Encapsulation Technologies: Pathways Toward Industrial Viability

Beyond the well‐established platforms of spray drying and conventional coacervation, several emerging encapsulation technologies have attracted sustained research attention over the past decade due to their capacity to achieve superior encapsulation efficiency, enhanced bioavailability, and more precise release control than conventional methods. These technologies including complex coacervation with plant‐based wall systems, electrospraying and electrospinning, inclusion complexation beyond β‐cyclodextrin, and novel nanoemulsion architectures occupy an important transitional space between laboratory innovation and commercial deployment. A critical evaluation of each technology’s industrial potential, current limitations, and realistic scale‐up pathways is essential if the field is to progress beyond the technology–implementation gap that this review has consistently identified.

### 11.1. Complex Coacervation: From Gelatin–GA to Plant Protein Systems

Complex coacervation remains one of the most technically capable encapsulation methods for high‐value applications, delivering encapsulation efficiencies of 85%–99% for hydrophobic cores such as EOs and omega‐3 fatty acids substantially higher than the 65%–80% typically achieved by spray drying for the same substrates [36]. The principal mechanism involves electrostatic interaction between oppositely charged biopolymers at controlled pH values, resulting in a dense coacervate phase that deposits around the core droplets to form mechanically robust microcapsules with excellent oxidative barriers [36, 74]. This superior performance is already reflected in the commercial success of coacervation‐based omega‐3 encapsulates in the premium supplement and infant formula markets, where the 40%–80% higher processing cost relative to spray drying is economically justified by shelf life extension from 12 to 18 months (spray drying) to 18–30 months (coacervation) [78].

However, the dominant coacervation system in current commercial‐use gelatin combined with GA faces three interconnected constraints that limit broader industrial adoption. First, gelatin is an animal‐derived protein that is incompatible with vegan, vegetarian, and halal or kosher product categories, restricting its use in a rapidly expanding market segment. Second, the high commercial cost of food‐grade GA (USD 8–15/kg at industrial volumes) contributes to the overall cost premium of coacervation relative to polysaccharide‐only spray‐dried systems [12, 36]. Third, as a naturally sourced material subject to seasonal and geographic variability, GA can exhibit batch‐to‐batch inconsistency that complicates quality assurance at industrial scale.

Recent research has convincingly demonstrated that associations of plant proteins with polysaccharides are capable of forming complex coacervates with functional properties comparable or superior to gelatin–GA systems, while offering reduced cost, broader sourcing availability, and alignment with sustainability‐driven consumer preferences [36, 39]. Muhoza et al. [36] showed that pea protein–pectin coacervates achieved encapsulation efficiencies of 87%–93% for EOs, with controlled‐release profiles across simulated gastrointestinal conditions. Liu et al. [39] further reported that Maillard conjugation between whey protein and MD improved interfacial stability and oxidative resistance compared to nonconjugated protein–polysaccharide mixtures, suggesting that chemically modified composite wall systems represent a productive direction for industrial optimization. Importantly, plant protein sources such as pea, lentil, soy, and sunflower protein isolates are available at costs of USD 3–7/kg at industrial scale substantially lower than gelatin (USD 5–12/kg) and comparable to GA in volume pricing, making plant protein‐based coacervation systems economically competitive for mainstream food applications, not only premium categories.

The principal obstacles to broad industrial adoption of plant–protein coacervation systems remain the sensitivity of coacervate formation to the isoelectric points and charge densities of the specific proteins used, the need for pH adjustment steps that increase process complexity, and the relatively limited published data on performance under realistic industrial processing conditions such as high‐shear mixing, spray atomization, and extended storage at fluctuating humidity. Addressing these barriers requires concerted industrial–academic collaboration to develop standardized coacervation protocols for plant protein systems and to generate the pilot‐scale performance data that are currently absent from the literature. Based on the decision framework proposed in Section 3, complex coacervation with plant–protein walls scores competitively estimated total framework score of 7.0–7.5 for omega‐3 delivery in functional beverages, compared to 8.03 for conventional spray drying and 7.41 for gelatin–GA coacervation, and could achieve parity with spray drying for premium applications once scalability limitations are addressed [26–28].

### 11.2. Electrospraying and Electrospinning: High‐Performance Platforms at the Scalability Frontier

Electrospraying and electrospinning represent two related but distinct electrostatic encapsulation modalities that have generated significant research interest for food bioactive delivery. In electrospraying, an electrostatic field is applied to a polymer solution containing the active compound, causing the liquid jet to break up into fine droplets that solidify on collection. Electrospinning produces continuous fibers rather than discrete particles, creating a nanofibrous matrix in which the bioactive is molecularly dispersed or nanoencapsulated. Both techniques are capable of producing structures in the 100 nm–5 μm range with morphological control that is difficult to achieve by conventional methods, and both have demonstrated effective encapsulation of thermolabile, volatile, and oxidation‐prone bioactives including carotenoids, polyphenols, EOs, and probiotics [52, 53, 66].

The demonstrated technical advantages of these technologies are significant. İnanç Horuz and Belibağlı [52] reported that electrospinning nanoencapsulation of lycopene from tomato peel extract in gelatin fibers increased water solubility by more than tenfold compared to the unencapsulated extract, while retaining 87% antioxidant activity over a 14‐day storage period, a marked improvement relative to conventional methods. Similarly, Berraquero‐García et al. [66] showed that electrospraying of bioactive peptides produced more uniform particle size distributions and higher encapsulation efficiencies than spray drying under comparable conditions, with improved controlled‐release profiles in simulated intestinal conditions. These performance advantages are consistent across the literature and represent a genuine technical advance rather than an incremental improvement.

Despite this impressive laboratory performance, the pathway from research to industrial deployment for both electrospraying and electrospinning faces substantial and to date largely unresolved challenges. The most fundamental constraint is throughput: conventional single‐needle electrospinning or electrospraying configurations produce material at rates of 0.001–0.1 kg/hour, compared to 100–1000 kg/hour for industrial spray dryers. Even advanced multineedle or needleless systems currently achieve maximum throughputs of 1–10 kg/hour, three to four orders of magnitude below what is required for mainstream food ingredient production [89, 112]. The economic implications are correspondingly severe: capital costs for industrial‐scale electrospinning systems capable of 100 kg/hour throughput are estimated at USD 2–8 million, and energy consumption of 18.5–28.0 MJ/kg product is three to five times higher than spray drying (Table 8), resulting in operating costs that preclude use in all but the highest‐value applications [89].

Additionally, the natural polymers most commonly used in food‐grade electrospraying—Ch, zein, and gelatin are relatively costly (USD 8–20/kg), may introduce off‐flavors at the concentrations required for effective encapsulation, and raise allergen or dietary restriction concerns in certain market contexts [112]. Regulatory pathways for nanostructured electrospun encapsulates remain unclear in several jurisdictions, as discussed in Section 10.4, further complicating commercialization.

Nevertheless, electrospraying and electrospinning occupy an important strategic niche in the encapsulation technology landscape. For ultrahigh‐value applications such as nutraceutical supplements, medical nutrition products, and functional packaging coatings where ingredient costs of USD 50–200/kg are routinely acceptable, the superior technical performance of electrospun or electrosprayed encapsulates can justify the associated production costs. Research priorities that would accelerate translation to industrial viability include the development of water‐soluble, sensory‐neutral biopolymers suitable for food‐grade electrospinning at lower concentrations and cost; the engineering of high‐throughput electrospinning systems with robust scale‐up predictability; and the generation of in situ performance data documenting the behavior of electrospun encapsulates under real food processing conditions such as mixing, baking, and high‐pressure homogenization [52, 53, 66, 89]. In the decision framework context, electrospun systems currently score approximately 4.5–5.5 out of 10 for mainstream food applications due to the economic and scalability limitations but could approach 7.0–7.5 for premium supplement or active‐packaging applications where technical performance criteria carry greater weight [26–28].

### 11.3. Inclusion Complexation Beyond β‐Cyclodextrin: Expanding the Host–Guest Encapsulation Platform

Cyclodextrin‐based inclusion complexation represents one of the most mechanistically elegant encapsulation strategies available to food technologists: The hydrophobic interior cavity of the cyclodextrin molecule captures apolar bioactive compounds through noncovalent host–guest interactions, effectively achieving molecular‐level encapsulation without the need for high‐temperature processing, organic solvents, or complex equipment [34, 35]. β‐Cyclodextrin has been the most widely studied host molecule and has achieved commercial adoption in flavor encapsulation and pharmaceutical applications; however, as discussed in Section 7.2.2, its regulatory status is geographically complex, with ADI restrictions in Europe (5 mg/kg body weight), GRAS status with usage category limitations in the United States, and premarket approval requirements in several Asian markets [34–36, 39].

The industrial potential of inclusion complexation has historically been constrained by the geometric specificity of the cyclodextrin cavity: The approximate internal diameter of β‐cyclodextrin (0.65 nm) accommodates only molecules of compatible molecular geometry, limiting the range of applicable core materials [34]. This geometric constraint effectively excludes macromolecular bioactives such as proteins, polysaccharides, and large polyphenolic compounds from encapsulation by cyclodextrin alone. However, recent research has expanded the practical scope of inclusion complexation through two complementary approaches: first, the use of modified cyclodextrins (hydroxypropyl‐β‐cyclodextrin, methyl‐β‐cyclodextrin, and acetylated derivatives) with modified cavity dimensions and improved aqueous solubility; and second, the development of cyclodextrin polymer networks and nanosponges that can accommodate a wider range of guest molecules than monomeric cyclodextrins [55, 56].

From an industrial perspective, inclusion complexation offers compelling advantages where applicable: It requires no thermal processing (important for thermolabile bioactives), produces dry, free‐flowing powders with excellent storage stability, does not require specialized encapsulation equipment beyond standard mixing vessels, and is cost‐effective for high‐volatility compounds such as flavors and EOs at commercial scale [34, 35]. Tian et al. [34] reviewed extensive evidence demonstrating that cyclodextrin inclusion complexes of EO components, phenolic antioxidants, and fat‐soluble vitamins exhibit significantly improved stability, water solubility, and controlled release relative to free compounds, with documented applications across flavor masking in nutraceuticals, antioxidant stabilization in functional beverages, and antimicrobial delivery in active‐packaging systems. The technique is particularly well‐suited for integration into the biorefinery paradigm discussed in Section 10.3, as cyclodextrins can potentially be produced from starch side‐streams using enzymatic cyclization, reducing raw material costs and supporting circular economy principles.

The key research priorities for advancing inclusion complexation toward broader industrial application include generation of comprehensive safety dossiers for modified cyclodextrin derivatives to support regulatory harmonization across major markets; development of continuous mixing processes for high‐throughput inclusion complex formation with quality‐by‐design validation; and systematic investigation of complex stability under the full range of food processing conditions particularly extrusion, baking, and high‐pressure treatment where existing data are sparse. The decision framework score for inclusion complexation is estimated at 6.5–7.5, depending on application, with economic viability being the primary constraining factor for nonflavor applications and regulatory compliance being the primary constraint for new geographical markets [26–28, 36, 39, 55, 56].

### 11.4. Novel Nanoemulsion and Multilayer Emulsion Architectures

Nanoemulsions oil‐in‐water or water‐in‐oil emulsions with droplet diameters below 200 nm have attracted growing industrial interest as delivery systems for lipophilic bioactives, omega‐3 fatty acids, and fat‐soluble vitamins due to their optical transparency (important in clear beverages), enhanced physical stability relative to conventional emulsions, and demonstrated improvements in oral bioavailability [81, 114]. Salvia‐Trujillo et al. [114] provided a comprehensive review of excipient nanoemulsions for improving oral bioavailability of bioactives, demonstrating that nanoemulsion droplet size reduction below 200 nm can increase the apparent bioavailability of lipophilic compounds by twofold to fivefold relative to macroemulsions through mechanisms including enhanced surface area for lipase activity, improved mucosal diffusion, and increased lymphatic uptake.

Multilayer emulsions, in which sequential electrostatic deposition of charged biopolymers creates two or more distinct interfacial layers around emulsion droplets, extend the functionality of conventional emulsions by providing enhanced oxidative barriers, stimulus‐responsive release, and improved stability under extreme processing conditions [81]. Jiménez‐Martín et al. [81] demonstrated that monolayered and multilayered emulsions for omega‐3 fatty acid microencapsulation by spray drying exhibited distinctly different oxidative stability profiles during storage, with the multilayer system providing significantly greater protection against lipid oxidation over a 12‐week accelerated storage period. Such systems are directly relevant to the commercial omega‐3 ingredient market discussed in Section 8.6, where oxidative stability is the primary technical challenge.

Industrially, nanoemulsions face the familiar nanoscale regulatory challenges discussed in Section 10.4, with droplets below 100 nm potentially triggering nanomaterial classification in certain jurisdictions. More practically significant for current industrial adoption is the energy intensity of nanoemulsion formation: high‐pressure homogenization (500–1500 bar) and microfluidiation are the most effective methods for achieving consistent sub‐200 nm droplet sizes at industrial scale, requiring capital investment of USD 200,000–1,000,000 for suitable equipment and energy inputs of approximately 2–5 MJ/kg product for the emulsification step [81, 92]. Operating within the decision framework, nanoemulsion systems score 5.5–7.0 for mainstream food applications, with the score range reflecting the considerable variation in application‐specific cost and regulatory constraints. For beverages and dairy products where optical clarity and superior bioavailability are marketable advantages, nanoemulsions represent a commercially viable near‐term option; for bakery and meat products where matrix turbidity is irrelevant and cost constraints are stringent, conventional spray‐dried emulsions remain preferable [26–28, 81, 114].

### 11.5. Hybrid and Multiscale Delivery Systems: The Emerging Frontier

The conclusion emerging from analysis of both established and emerging encapsulation technologies is that no single technology optimally satisfies all four dimensions of the decision framework: technical performance, economic viability, regulatory compliance, and environmental sustainability across the full spectrum of food applications. This recognition is driving increasing research interest in hybrid and multiscale delivery systems, in which the complementary advantages of different encapsulation architectures are combined within a single functional system [19].

Representative hybrid approaches include the encapsulation of nanostructured lipid carriers within spray‐dried microcapsule matrices to combine the bioavailability enhancement of nanoparticles with the scalability and regulatory acceptability of spray‐dried microcapsules; the incorporation of cyclodextrin inclusion complexes within alginate or protein gel beads to provide both molecular‐level and particulate‐level protection for volatile or oxidation‐sensitive compounds; and the coencapsulation of bioactives with natural antioxidants (tocopherols, rosemary extract) within multilayer emulsion systems to simultaneously address oxidative stability and controlled release [19, 36, 81]. These hybrid systems represent the direction that the field must take if encapsulation technology is to fullfil its potential as a platform for next‐generation food innovation rather than remaining an incremental improvement on existing ingredient stabilization approaches.

Realizing the industrial potential of hybrid encapsulation systems requires addressing two prerequisites that are currently underdeveloped in the literature. The first is the systematic characterization of the interactions between the different encapsulation components in hybrid systems interactions that can be synergistic (as when nanoparticulate dispersibility enhances microparticle reconstitution) or antagonistic (as when ionic cross‐linking agents from one encapsulation step destabilize the wall material of another). The second prerequisite is the development of scalable manufacturing processes for hybrid systems that do not merely concatenate the cost and complexity of two independent encapsulation processes. Both prerequisites require sustained investment in process engineering and pilot‐scale validation that extends well beyond what is currently reported in the academic literature, reinforcing the central argument of this review that meaningful progress in food encapsulation requires not only technical innovation but a fundamental reorientation toward performance‐based, industrially grounded evaluation frameworks [1, 6, 19].

## 12. Conclusion

Encapsulation technology can be defined as a fundamental and facilitating platform in the present food science, whereby it offers effective means of stabilizing, protecting, and controlled delivery of bioactive compounds in various food systems. In place of being a single purpose end‐use innovation, encapsulation supports a wide range of uses, such as the design of functional foods, delivery of nutraceuticals, ingredient fortification, and active and intelligent packaging. In this context, edible films/coatings are to be perceived as an essential area of applications of encapsulation, in which encapsulated bioactivities are delivered into biodegradable verses to improve food preservation, safety, and quality via a controlled‐release system. This review shows in critically that the current focus on encapsulation efficiency as being alone is not adequate to extrapolate to actual functionality. High encapsulation efficiency measured under laboratory conditions does not guarantee improved shelf stability, sensory acceptability, and sensory activities in a complex food system, industrial processing stresses, and gastrointestinal initials. The compatibility between the wall core, kinetic release, destabilization by the matrix, and processing history are some of the factors that determine the functional success of encapsulated systems. It therefore follows that to promote encapsulation between experimental research and industrial viable solutions, performance‐focused measurement of evaluation, as opposed to method‐driven optimization, is critical. Relative discussion of microencapsulation and nanoencapsulation points out that although there is the increasing interest and attraction of nanoscale systems, microencapsulation is the most frequent and viable route to food industry usages because of its acceptance in regulations, strength, low cost of production, as well as scalability. Although nanoencapsulation has some benefits of modifiable release behavior and access to a larger surface area, aggregation issues, oxidative degradation, uncertain regulatory status, and high cost of production all are obstacles to its extensive application at present. The future encapsulation schemes will primarily, thus, be based on hybrid or rather multiscale systems combining the nanostructural functional benefits with the microscaled carrier industrial feasibility. The choice of materials to be used as walls comes as a determining element of encapsulation. Each polysaccharide, proteins, lipids, and composite systems have different advantages and weaknesses depending on the type of core material being used and the application. The use of composite and conjugated wall systems especially protein–polysaccharide systems is promising with regard to enhancing oxidative stability, interfacial strength, and dictated release characteristics in complex food situations. Nonetheless, issues of formulation complexity and batch‐to‐batch variability are impediments to scale, whereby standardized design frameworks and scalable dictates need to be developed. Applicatively, encapsulation has shown great potential in beverages and bakery, dairy, meat, and functional food industries through improvement of ingredient stability, concealing undesirable taste and delivery of sensitive bioactives such as vitamins, probiotics, EOs, and plant extracts. Within the framework of edible films and coating application, encapsulation is an important component in the creation of active‐packaging systems where food preservatives are substituted by synthetic preservatives, increasing shelf and food safety, and prolonging shelf life. The following applications demonstrate how flexible the concept of encapsulation is as a technology platform as opposed to a separate processing mechanism. To summarize, the future development of encapsulation in food systems is pegged on the incorporation of both the functionality of performance and a combination of economic viability, legislation and compliance, and sustainable environment. There should be the consideration of life‐cycle assessment, energy efficiency, and material circularity in addition to encapsulation efficiency and release performance. The approach of prioritizing research studies on the development of decision‐based design structures including considerations of real‐life conditions of processing and end‐user needs will be highly critical in the realization of the full industrial potential of encapsulation technology. Encapsulation may further develop as an innovative instrument of the next generation of food innovation, by going beyond technique‐based optimization and adopting application‐relevant performance measures.

## Author Contributions

Sumon Islam: writing–original draft, data curation, formal analysis, investigation, and methodology. Md. Hassan Bin Nabi: writing–original draft, writing–review and editing, formal analysis, software, methodology, investigation, conceptualization, validation, and visualization. Iftekhar Ahmad: writing–review and editing, formal analysis, and supervision. Wahidu Zzaman: writing–review and editing, conceptualization, supervision, and project administration.

## Funding

This research did not receive any specific grant from funding agencies in the public, commercial, or not‐for‐profit sectors.

## Disclosure

All authors participated in editing the article and agreed to the published version of the manuscript.

## Conflicts of Interest

The authors declare no conflicts of interest.

## Data Availability

The data that support the findings of this study are available from the corresponding author upon reasonable request.
